# Effect of a Peer-Led Behavioral Intervention for Emergency Department Patients at High Risk of Fatal Opioid Overdose: A Randomized Clinical Trial

**DOI:** 10.1001/jamanetworkopen.2022.25582

**Published:** 2022-08-09

**Authors:** Francesca L. Beaudoin, Brendan P. Jacka, Yu Li, Elizabeth A. Samuels, Benjamin D. Hallowell, Alyssa M. Peachey, Roxxanne A. Newman, Mackenzie M. Daly, Kirsten J. Langdon, Brandon D. L. Marshall

**Affiliations:** 1 Brown University School of Public Health, Providence, Rhode Island; 2 Department of Emergency Medicine, Alpert Medical School of Brown University, Providence, Rhode Island; 3 Rhode Island Department of Health, Providence; 4 Rhode Island Department of Behavioral Healthcare, Developmental Disabilities and Hospitals, Providence; 5 Department of Psychiatry, Rhode Island Hospital, Providence; 6 Department of Psychiatry and Human Behavior, Alpert Medical School of Brown University, Providence, Rhode Island; 7 Brown-Lifespan Center for Digital Health, Brown University, Providence, Rhode Island

## Abstract

**Importance:**

Fatal and nonfatal opioid overdoses are at record levels, and emergency department (ED) visits may be an opportune time to intervene. Peer-led models of care are increasingly common; however, little is known about their effectiveness.

**Objective:**

To evaluate the effect of a peer-led behavioral intervention compared with the standard behavioral intervention delivered in the ED on engagement in substance use disorder (SUD) treatment within 30 days after the ED encounter.

**Design, Setting, and Participants:**

This randomized clinical trial recruited 648 patients from 2 EDs from November 15, 2018, to May 31, 2021. Patients were eligible to participate if they were in the ED for an opioid overdose, receiving treatment related to an opioid use disorder, or identified as having had a recent opioid overdose.

**Interventions:**

Participants were randomly assigned to receive a behavioral intervention from a certified peer recovery specialist (n = 323) or a standard intervention delivered by a hospital-employed licensed clinical social worker (n = 325). A certified peer recovery specialist was someone with at least 2 years of recovery who completed a 45-hour training program and had 500 hours of supervised work experience. After the ED intervention, the certified peer recovery specialists offered continued contact with participants for up to 90 days.

**Main Outcomes and Measures:**

The primary outcome was receipt of SUD treatment within 30 days of enrollment, assessed with deterministic linkage of statewide administrative databases. Treatment engagement was defined as admission to a formal, publicly licensed SUD treatment program or receipt of office-based medication for opioid use disorder within 30 days of the initial ED visit.

**Results:**

Among the 648 participants, the mean (SD) age was 36.9 (10.8) years, and most were male (442 [68.2%]) and White (444 [68.5%]). Receipt of SUD treatment occurred for 103 of 323 participants (32%) in the intervention group vs 98 of 325 participants (30%) in the usual care group within 30 days of the ED visit. Among all participants, the most accessed treatments were outpatient medication for opioid use disorder (buprenorphine, 119 [18.4%]; methadone, 44 [6.8%]) and residential treatment (44 [6.8%]).

**Conclusions and Relevance:**

Overall, this study found that a substantial proportion of participants in both groups engaged in SUD treatment within 30 days of the ED visit. An ED-based behavioral intervention is likely effective in promoting treatment engagement, but who delivers the intervention may be less influential on short-term outcomes. Further study is required to determine the effects on longer-term engagement in SUD care and other health outcomes (eg, recurrent overdose).

**Trial Registration:**

ClinicalTrials.gov Identifier: NCT03684681

## Key Points

**Question:**

Can peer-led behavioral interventions in the emergency department for patients who have had a recent opioid overdose increase short-term treatment engagement after the emergency department visit?

**Findings:**

In this randomized clinical trial of 648 emergency department patients at high risk of opioid overdose, there was no difference in treatment engagement within 30 days of the visit for participants who received a peer-led intervention vs those who received a standard behavioral intervention by a clinical social worker (32% vs 30%).

**Meaning:**

An emergency department–based behavioral intervention is likely effective in promoting treatment engagement, but who delivers the intervention may be less influential on short-term outcomes.

## Teaser

This randomized clinical trial evaluates whether peer-led behavioral interventions for emergency department patients at high risk of opioid overdose increase treatment engagement within 30 days.

## Introduction

In 2021, more opioid overdose deaths were recorded in the United States than in any prior year,^1,2^ and opioid-related visits to US emergency departments (EDs) increased 4-fold from 2008 to 2018.^3^ Patients admitted to an ED for an overdose are at greatly elevated risk for a subsequent nonfatal overdose and overdose death.^4,5,6^ Thus, the ED encounter has been presented as both a critical and opportune time to prevent recurrent opioid overdoses and death through increased uptake in addiction treatment and provision of other harm reduction and recovery services.^7^ However, to our knowledge, there is limited evidence on the most effective ways to promote engagement in treatment after discharge from an ED.

As 1 potential solution, peer-led behavioral interventions delivered by certified peer recovery specialists have been implemented in EDs throughout the country. Peer support has a well-established role in community-based treatment and recovery approaches,^8,9,10,11,12,13,14,15^ but only more recently has it been integrated into ED staffing models. To our knowledge, there is a paucity of studies rigorously evaluating peer recovery support services, and to date, none have been conducted in the ED setting.^16,17^ Pilot programs of ED-based peer support have shown promise in increasing access to substance use disorder (SUD) treatment and care,^18,19,20^ but it is still unknown whether a peer-led approach is more effective than other approaches. We sought to compare the effectiveness of a peer-led behavioral intervention vs a standard one to increase treatment uptake among ED patients who were at greatest risk of accidental drug-related death.

## Methods

### Trial Design

This study was a parallel-group, randomized clinical trial in which patients at high risk for opioid overdose who presented to the ED were randomly assigned 1:1 to receive a behavioral intervention from either a certified peer recovery specialist (“peer”) or a licensed clinical social worker (“social worker”). At the outset, we had also intended to enroll a companion cohort of control participants who refused any intervention (as part of the study or clinical care), but this was closed for futility 6 months after launch. Participants completed baseline assessments and consented to review of electronic health records and linkage to state administrative data (including substance use treatment records) to objectively assess the study outcomes. The full study protocol (NCT03684681) has been published elsewhere (trial protocol and statistical analysis plan in Supplement 1).^21^ Ethical approval was obtained from the Lifespan institutional review board. All participants provided written informed consent. The study followed the Consolidated Standards of Reporting Trials (CONSORT) reporting guideline.

### Setting, Timeline, and Population

The study was conducted in 2 EDs in Rhode Island, one in an academic tertiary care hospital and the other in a high-volume, community-based affiliate. The 2 EDs combined have more than 170 000 adult visits annually and receive approximately 50% of all the overdose-related ED visits in Rhode Island.^22^ Recruitment occurred from November 15, 2018, to May 31, 2021. There was a brief pause in enrollment during April and May 2020 due to the COVID-19 pandemic. A consecutive sample of patients presenting to the ED (24 hours per day, 7 days per week) was assessed for eligibility by study research staff.

To be eligible, participants must have met 1 of the following criteria: they were in the ED for an opioid overdose at the time of the visit, they received treatment related to an opioid use disorder (OUD) (eg, infectious complication or opioid withdrawal) at the time of the visit, or they were identified as having had an opioid overdose within the previous 12 months (self-report or electronic health record review). The presence of an opioid overdose at the ED visit was determined by the treating physician and was generally defined as (1) the presence of decreased levels of consciousness or respiratory depression, (2) occurring after the consumption of opioids, and (3) resolving after the administration of naloxone. Potential participants were deemed ineligible if they were previously enrolled in the trial, were in police custody or incarcerated, were pregnant, predominantly lived outside of Rhode Island, or were unable to provide informed consent.

### Interventions

The trial was conducted using the existing infrastructure. Participants received a behavioral intervention delivered by either a peer or a social worker. Before the trial started, it was standard of care to receive a behavioral intervention in the ED after an opioid overdose, but not necessarily for OUD in general. Both social workers and peers were available to provide behavioral interventions in the ED, but this consultation was performed at the discretion of the treating physician. In accordance with quality data, approximately half of all patients with a visit for opioid overdose received a behavioral intervention, with approximately equal numbers consulting a social worker vs a peer.

Social workers were employed by the recruitment sites (hospitals). They were chosen as the active comparator group because they are often extant staff within hospital systems. A community-based organization in Rhode Island (Anchor Recovery Community Center^18,23,24^) provided the certified peer recovery specialist staff for the study. Individuals employed as a certified peer recovery specialist have had at least 2 years of recovery and completed a 45-hour training program with supervised work experience (500 hours). The training program is based on the trauma-informed model of care and the transtheoretic model of behavior change, with focus on wellness and recovery, motivational interviewing, mentoring, and advocacy.

Both groups of interventionists underwent training before study initiation and then regular, standard training according to their standard practice. Because this trial was an effectiveness study, we did not monitor or ensure the fidelity of the interventions throughout the study. Both groups of interventionists were trained to administer a range of evidence-based interviewing and intervention techniques to support patients who are attending the ED for an opioid overdose or who are identified as having an OUD. A key distinction is that social workers rely on clinical experience and social work theory and practice, whereas peers draw on lived experience of SUD and recovery to inform the intervention. Throughout the study, both the peers and social workers were expected to respond to a consultation within 30 minutes of request. Both social workers and peer interventionists aimed to address the immediate and long-term needs of clients, such as take-home naloxone and SUD treatment access, but also other factors, such as housing and transportation. The social work intervention was delivered as a onetime, generally brief intervention without contact after ED discharge. Although there was no time limit to the intervention, most lasted less than 30 minutes. Peers also delivered a brief intervention in the ED, but they also continued contact after ED discharge. After the ED visit, the peers followed protocol and initiated and continued contact with individuals for 3 months (daily for the first 10 days and then weekly unless services were decreased) in accordance with their standard practice.^23^ On review of quality data, peers were able to make contact with 85% of participants within the first 10 days after the ED visit. Participants could opt in to ongoing peer-support services beyond 3 months from the community-based organization. Participants in both groups received usual clinical care services, including naloxone, electronic referrals to SUD treatment, and, if interested, prescriptions for medications for OUD (mainly buprenorphine).

### Outcomes and Measures

The primary outcomes of the trial were engagement with a formal SUD treatment program within 30 days of the initial ED visit and recurrent ED visits for a suspected opioid overdose during 18 months. A major strength of this study is that both end points were assessed via linkage to statewide administrative databases, as outlined later. We report herein on the first end point (treatment engagement) because participant follow-up for the second primary end point (ED visit for opioid overdose) is still under way and will not be completed until November 2022. There was not an a priori stopping rule based on the first end point, and analysis for the second end point is expected to commence in early 2023. Treatment engagement was defined as admission to a formal, publicly licensed SUD treatment program or receipt of office-based medication for OUD within 30 days of the initial ED visit—specifically, an inpatient detoxification program, outpatient or residential treatment programs, or outpatient medication for OUD (ie, methadone, buprenorphine, or naltrexone).^25^ The outcome was assessed with deterministic linkages to the Behavioral Health Online Database, a state database containing information on all admission and discharge events of clients of all behavioral health care organizations licensed by the Rhode Island Department of Behavioral Healthcare, Developmental Disabilities and Hospitals.^17^ Participants who received buprenorphine or naltrexone in office-based settings were identified via linkage to the state’s prescription drug monitoring program, maintained by the Rhode Island Department of Health.^26^ As detailed in the study protocol, a comprehensive biobehavioral questionnaire was administered at enrollment and included questions about demographic characteristics (eg, self-reported race and ethnicity, gender identity, and sex at birth) and substance use (eg, past use, SUD treatment, and self-reported readiness for change).^21^

### Sample Size

In accordance with quality improvement data, we assumed that 23 of 325 participants (7%) randomly assigned to receive a behavioral intervention from a social worker would enroll in a formal SUD treatment program within 30 days of their ED visit. A sample size of 650 participants would provide at least 80% power to detect a 2-fold increase in treatment engagement (ie, 14%) among participants assigned to receive a behavioral intervention from a peer recovery specialist; this increase was identified as an appropriate benchmark by a group of key stakeholders that included clinicians, policy makers, and patient advocacy representatives.

### Randomization and Blinding

Participants were randomly assigned to 1 of the 2 study groups (1:1) according to permuted block sizes and stratified on study site, sex, and whether the visit was for a current overdose. The randomization schedule was maintained by the study data manager not involved with recruitment or the final analyses. Participants and physicians were not blinded to their intervention assignment; however, the main statistical analyses were performed by a blinded statistician.

### Statistical Analysis

To determine the success of randomization, we described and compared (eg, using the χ^2^ test or the *t* test) the distribution of baseline characteristics stratified by the study group. For evaluation of our primary end point, we used an intention-to-treat approach to estimate the average treatment effect. We compared the differences in the primary end point between treatment groups using the χ^2^ test. Exploratory subgroup analyses were performed to examine enrollment in any SUD program according to reason for ED visit (ie, opioid overdose–related vs nonoverdose-related visit) and SUD treatment exposure in the 30 days before enrollment. Specifically, 4 log-binomial regression analyses were conducted: without adjustment (model 1); with adjustment for block randomization variables (ie, site, aged <50 years, and sex at birth [model 2]); an interaction term between intervention group and reason for ED visit (model 3) with adjustment for randomization variables; and an interaction term between intervention group and prior or curent SUD treatment exposure (model 4). The results from models 3 and 4 were summarized according to established guidelines for reporting interaction.^27^ All analyses were conducted with SAS, version 9.4 (SAS Institute), with a priori levels of significance set to .05 and hypothesis tests conducted as 2 sided.

## Results

For the 648 study participants, the mean (SD) age was 36.9 (10.8) years. The study groups were balanced with respect to baseline demographic characteristics (Table 1) except for age, for which participants in the social worker group (mean [SD] age, 37.2 [11.1] years) were statistically older than those in the peer group (mean [SD] age, 36.4 [10.5] years). Overall, 442 of the participants identified as male (68.2%) and 206 as female (31.8%). A total of 17 were American Indian or Alaska Native (2.6%); 3 were Asian (0.5%); 39 were Black, African, Haitian, or Cape Verdean (6.0%); 107 were Hispanic (16.5%); 61 were of mixed race, biracial, or multiracial (9.4%); 3 were Native Hawaiian or Other Pacific Islander (0.5%); 541 were non-Hispanic (83.5%); 444 were White (68.5%); and 62 (9.6%) were of other race (race and ethnicity were self-reported, and no further level of detail for “other” was available). More than half of enrolled participants (353 [54.5%]) were attending the ED for reasons other than opioid-related overdose, whereas slightly less than half (293 [45.4%]) were identified as having had a recent overdose. Half of participants had reported injecting drugs in their lifetime. A large proportion of participants (500 of 627 [79.7%]) reported adverse social determinants of health, including unstable housing, current unemployment (450 [69.4%]), and a current monthly income of $0 (158 [24.4%]).

**Table 1.** Baseline Characteristics of the Participants Enrolled in the Trial, Stratified by Study Group

Characteristic	Participants, No. (%)		
	Total	Social worker	Peer
No.	648	325	323
Age, mean (SD), y	36.9 (10.8)	37.2 (11.1)	36.4 (10.5)
Sex at birth			
Male	442 (68.2)	224 (68.9)	218 (67.5)
Female	206 (31.8)	101 (31.1)	105 (32.5)
Gender identity			
Female	202 (31.2)	99 (30.5)	103 (31.9)
Male	439 (67.7)	223 (68.6)	216 (66.9)
Transgender	1 (0.2)	1 (0.3)	0
Race			
American Indian or Alaska Native	17 (2.6)	9 (2.8)	8 (2.5)
Asian	3 (0.5)	2 (0.6)	1 (0.3)
Black, African, Haitian, or Cape Verdean	39 (6.0)	20 (6.2)	19 (5.9)
Mixed, biracial, or multiracial	61 (9.4)	27 (8.3)	34 (10.5)
Native Hawaiian or Other Pacific Islander	3 (0.5)	2 (0.6)	1 (0.3)
White	444 (68.5)	226 (69.5)	218 (67.5)
Other^a^	62 (9.6)	29 (8.9)	33 (10.2)
Did not know or refused to answer	13 (2.0)	7 (2.2)	6 (1.9)
Hispanic ethnicity			
Non-Hispanic	541 (83.5)	276 (84.9)	265 (82.0)
Hispanic	107 (16.5)	49 (15.1)	58 (18.0)
Overdose as reason for enrollment visit			
No	353 (54.5)	180 (55.4)	173 (53.6)
Yes	293 (45.4)	144 (44.4)	149 (46.3)
Current health insurance coverage			
Yes	576 (90.6)	289 (88.9)	287 (88.9)
No	48 (7.4)	25 (7.7)	23 (7.1)
Did not know or refused to answer	12 (1.9)	5 (1.5)	7 (2.2)
Unstable housing			
No	192 (29.6)	94 (28.9)	98 (30.3)
Not in past 6 mo	158 (24.4)	86 (26.5)	72 (22.3)
Past 6 mo	283 (43.7)	138 (42.5)	145 (44.9)
Currently employed full time or part time			
No	450 (69.4)	224 (68.9)	226 (70.0)
Part time or full time	181 (27.9)	95 (29.2)	86 (26.6)
Did not know or refused to answer	6 (0.9)	2 (0.6)	4 (1.2)
Current monthly income, $			
0	158 (24.4)	81 (24.9)	77 (23.8)
<1-500	88 (13.6)	42 (12.9)	46 (14.2)
501-1500	193 (29.8)	103 (31.7)	90 (27.9)
1501-3000	99 (15.3)	49 (15.1)	50 (15.5)
>3000	55 (8.5)	24 (7.4)	31 (9.6)
Did not know or refused to answer	44 (6.8)	22 (6.8)	22 (6.8)
Plans to change drug use			
Yes	562 (86.7)	281 (86.5)	281 (87.0)
No	37 (5.7)	17 (5.2)	20 (6.2)
Maybe	17 (2.6)	10 (3.1)	7 (2.2)
Did not know or refused to answer	16 (2.5)	8 (2.5)	8 (2.5)
When do you think you might do so?^b^			
More than 12 mo from now	6 (0.9)	4 (1.2)	2 (0.6)
In the next 6 to 12 mo	11 (1.7)	8 (2.5)	3 (0.9)
In the next 1 to 6 mo	17 (2.6)	11 (3.4)	6 (1.9)
In the next 30 d	53 (8.2)	23 (7.1)	30 (9.3)
Today	472 (72.8)	235 (72.3)	237 (73.4)
Motivation and readiness for treatment, mean (SD)^c^	46.6 (7.5)	46.6 (7.3)	46.6 (7.7)
Addiction treatment^d^			
Never	133 (20.5)	61 (18.8)	72 (22.3)
Not currently	314 (48.5)	152 (46.8)	162 (50.2)
Currently	178 (27.5)	100 (30.8)	78 (24.1)
Ever received methadone treatment^d^			
Yes	253 (39.0)	162 (49.8)	127 (39.3)
No	395 (61.0)	199 (61.2)	196 (60.7)
Ever received buprenorphine treatment^d^			
Yes	236 (36.4)	107 (32.9)	129 (39.9)
No	412 (63.6)	218 (67.1)	194 (60.1)
Ever experienced barrier to treatment access^d^			
Yes	226 (34.9)	113 (34.8)	113 (35.0)
No	401 (61.9)	203 (62.5)	198 (61.3)
Did not know or refused to answer	12 (1.9)	5 (1.5)	7 (2.2)
Ever injected drugs^d^			
Yes	333 (51.4)	170 (52.3)	163 (50.5)
No	261 (40.3)	132 (40.6)	129 (39.9)
Did not know or refused to answer	11 (1.7)	6 (1.8)	5 (1.5)

^a^ Race and ethnicity were self-reported, and no further level of detail for “other” was available.

^b^ If plans to change were endorsed.

^c^ Data missing for 43 participants.

^d^ Self-reported.

### Recruitment

Initial screening of electronic health records identified 10 815 potentially eligible individuals attending the ED from November 15, 2018, to May 31, 2021. After ongoing detailed review and application of electronic health record eligibility criteria, 2236 individuals (20.7%) met the eligibility criteria and were approached to participate (Figure). An additional 429 individuals were referred by their treating physician. Of the 2665 individuals who completed in-person screening, 658 (24.7%) consented to participate, and 648 (24.3%) were randomly assigned to study groups (social worker, 325 participants; peer, 323 participants). Most participants (627 of 648 [96.8%]) completed the intervention; of those who did not, 2 (1 in each group) did not receive any intervention, and the remaining 19 met with the interventionist but left the ED before completing the interaction. The most-cited reason for not completing the intervention was “not wanting to wait.” In alignment with intention-to-treat principles, all randomized participants were analyzed (n = 648) regardless of whether the intervention was completed.

**Figure.**
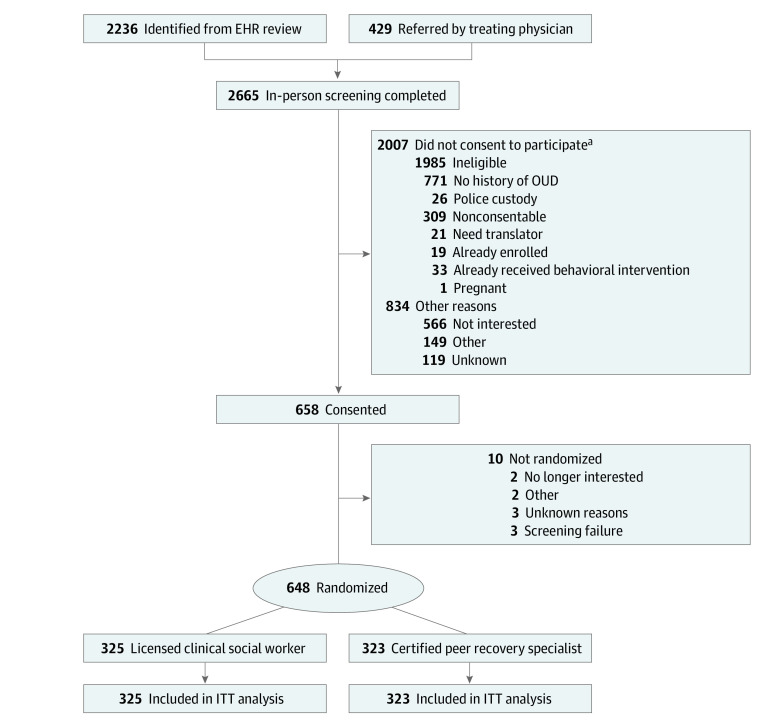
Trial Participant Flow Diagram EHR indicates electronic health record; ITT, intention-to-treat; and OUD, opioid use disorder. ^a^More than 1 response possible.

Among the participants who reported plans to change their drug use (562 of 648 [86.7%]), 472 (72.8%) indicated they might do so that day. Most participants had reported previous experience with treatment for an SUD (497 of 630 [78.9%]), with a similar proportion reporting experience with methadone (253 [39.0%]) and buprenorphine (236 [36.4%]). Approximately one-quarter of participants (185 [28.5%]) reported receiving treatment at enrollment. Barriers to access were common among participants, with one-third (226 [34.9%]) reporting ever experiencing difficulty initiating a treatment program. In addition, 269 participants (41.5%) were interested in receiving a referral for an SUD treatment program at the visit, and 255 (39.4%) were very or somewhat interested in starting medication for OUD. Eighty participants (12.3%) were interested in starting buprenorphine at the visit.

### Primary Outcome

We observed no significant difference between the 2 groups in enrollment in an SUD treatment program within 30 days of the ED visit, with 98 of 325 participants (30%) and 103 of 323 participants (32%) in the social worker and peer groups experiencing this outcome, respectively (eTable 1 in Supplement 2). The relative risk of the outcome was not significantly different between the 2 groups in the unadjusted log-binomial model or when adjusting for block randomization variables (eTable 1 in Supplement 2). Results were similar when the interaction between study group and reason for the ED visit (overdose or nonoverdose visit) was examined, with no significant interaction between them (Table 2 and eTable 2 in Supplement 2). Individuals with prior SUD treatment were more likely to engage in post-ED treatment in both intervention groups (social worker: relative risk, 2.51 [95% CI, 1.86-3.40]); peer: relative risk, 2.51 [95% CI, 1.86-3.50]) (Table 3 and eTable 2 in Supplement 2). However, there was no significant interaction between study group and history of SUD treatment before enrollment; in other words, there was no difference in outcomes between study groups after accounting for SUD treatment before enrollment.

**Table 2.** Interaction Between Reason for Emergency Department Visit and Study Group on the Risk of 30 Days of Enrollment in the Trial^a^

Study group	Nonopioid overdose–related visit			Opioid overdose–related visit			Opioid overdose–related visit (yes vs no) within strata of study groups, RR (95% CI)	*P* value
	No. enrolled in SUD program (yes/no)	RR (95% CI)	*P* value	No. enrolled in SUD program (yes/no)	RR (95% CI)	*P* value		
Social worker	59/121	1 [Reference]	NA	38/106	0.81 (0.57-1.14)	.22	0.81 (0.57-1.14)	.22
Peer	60/113	1.05 (0.78-1.40)	.75	43/106	0.88 (0.63-1.22)	.44	0.84 (0.61-1.16)	.28
Study groups (peer vs social worker) within strata of opioid overdose–related visit	NA	1.05 (0.78-1.40)	.75	NA	1.09 (0.75-1.58)	.65	NA	NA

Abbreviations: NA, not applicable; RR, relative risk; SUD, substance use disorder.

^a^ Log binomial regression models adjusted for the following randomization blocking variables: age category (<50 years), sex at birth, and hospital site.

**Table 3.** Interaction Between Prior Substance Use Disorder Treatment and Study Group on the Risk of Enrollment in Substance Use Disorder Program Within 30 Days of Enrollment in the Trial^a^

Study group	No prior treatment exposure			Prior treatment exposure			Prior treatment exposure (yes vs no) within strata of study groups, RR (95% CI)	*P* value
	No. enrolled in SUD program (yes/no)	RR (95% CI)	*P* value	No. enrolled in SUD program (yes/no)	RR (95% CI)	*P* value		
Social worker	53/185	1 [Reference]	NA	45/42	2.31 (1.69-3.16)	<.001	2.31 (1.69-3.16)	<.001
Peer	52/182	1.00 (0.71-1.40)	>.99	51/38	2.51 (1.86-3.39)	<.001	2.51 (1.86-3.40)	<.001
Study groups (peer vs social worker) within strata of prior treatment exposure, RR (95% CI)	NA	1.00 (0.71-1.40)	>.99	NA	1.09 (0.83-1.42)	.55	NA	NA

Abbreviations: NA, not applicable; RR, relative risk; SUD, substance use disorder.

^a^ Log binomial regression models adjusted for the following randomization blocking variables: age category (<50 years), sex at birth, and hospital site.

One-quarter of the 648 participants received medication within 30 days of the index visit, either buprenorphine (119 [18.4%]) or methadone (44 [6.8%]), for OUD within 30 days of enrollment (Table 4). Smaller proportions of participants accessed acute or crisis stabilization units (48 [7.4%]), residential treatment (44 [6.8%]), and an inpatient detoxification program (28 [4.3%]). There was little difference in receipt of individual SUD treatment types across study groups.

**Table 4.** Treatment Initiation in Selected Substance Use Disorder Program Within 30 Days of Enrollment in the Trial

Program type	Participant, No. (%)		
	Total	Social worker	Peer
No.	648	325	323
Substance use disorder program			
Detoxification	28 (4.3)	10 (3.1)	18 (5.6)
Methadone for opioid use disorder	44 (6.8)	18 (5.5)	26 (8.0)
Intensive outpatient	3 (0.5)	2 (0.6)	1 (0.3)
Outpatient	6 (0.9)	2 (0.6)	4 (1.2)
Residential treatment	44 (6.8)	22 (6.8)	22 (6.8)
Buprenorphine	119 (18.4)	63 (19.4)	56 (17.3)
Mental health program^a^			
Acute stabilization unit or crisis stabilization unit	48 (7.4)	28 (8.6)	20 (6.2)
Community support program	4 (0.6)	2 (0.6)	2 (0.6)
Outpatient	7 (1.1)	2 (0.6)	5 (1.5)

^a^ Not included in primary outcome.

## Discussion

In this large, randomized clinical trial of 2 different behavioral interventions in the ED for patients at high risk of opioid overdose, there was no difference in SUD treatment uptake within 30 days of the ED visit between participants who received a peer-led intervention and those who had an intervention delivered by a licensed clinical social worker. To our knowledge, this trial represents the first systematic evaluation of behavioral interventions delivered in the ED for patients at high risk of opioid overdose and is also the first to examine whether a peer-based model is more effective than a brief onetime intervention delivered by a clinical social worker.

Substance use disorder treatment uptake within 30 days of the ED visit was high in both study groups compared with that in previous studies,^28^ with nearly one-third of patients accessing treatment within this time frame. Moreover, this proportion may underrepresent the total number of participants who accessed treatment because it would not reflect certain services (eg, out-of-state or private programs not licensed by the Department of Behavioral Healthcare, Developmental Disabilities and Hospitals). Since the introduction of the standards of care for patients presenting for opioid overdose in Rhode Island, 50% to 75% of eligible patients received a referral to SUD treatment.^29^ There are limited data on the historical rates of SUD uptake after an ED visit, although referrals to SUD treatment during an ED visit have previously ranged from 9% to 20% in our setting.^30^ In the review of quality improvement data from the Rhode Island Department of Health (from April 2016 to March 2021), 22.5% of all patients who visited 1 of the 2 study EDs after an opioid overdose subsequently engaged in treatment in the 30 days after their visit. This estimate uses the same data sources and outcome definition as this study and also includes some of the study participants who were attending the ED after an overdose. It is likely, then, that the rates of engagement observed in our study are higher than previously noted, supporting the idea that a behavioral intervention in the ED is an opportunity to direct patients to SUD treatment, regardless of the person who delivers it.

The ED visit for overdose has often been suggested as a teachable moment^31^; however, this concept was born out of early literature in other disease states (eg, smoking cessation counseling after a new cancer diagnosis). In addition, previous studies of ED-based behavioral interventions for SUD have had mixed results, calling into question this approach.^32^ It is also unknown whether the overdose visit itself is the most appropriate opportunity for many patients; the psychosocial and physiologic circumstances of the overdose event may actually make it a difficult time to successfully engage patients. Indeed, patients with OUD may not perceive the ED as a suitable location for OUD treatment, in part because of prior experiences of stigmatization and discrimination by ED staff, minimization of their medical needs, and inadequate resources for EDs to provide treatment for OUD.^33^ However, in our subgroup analysis, there were no meaningful differences in treatment engagement after the ED visit between individuals who were there after an overdose and those who were there for other reasons but who were identified as having had a recent overdose or an OUD-related visit. This suggests that continued engagement after an overdose visit is important but that other patients at high risk should also be considered candidates for a behavioral intervention.

In a subgroup analysis, we observed that individuals with a treatment history were more than twice as likely to engage in treatment compared with those without one. This finding was consistent across both intervention groups. However, a significant proportion of individuals without prior treatment exposure still engaged in treatment (143 of 648 [22%]), suggesting that, although individuals with prior treatment may be more amenable to reengagement, the lack of treatment history should not discount a willingness to engage. To reflect the actual situation that would be encountered in the ED, we chose to allow people to enter the study regardless of treatment history.

Although our study findings describe the average effects of treatment, there may be individual or subgroup causal effects not identified by this study design. Patient perspectives and experiences are also not measured in this study, and it is likely that some patients respond better to a peer than to a social worker. Future studies might examine predictors of treatment response within each study group to begin to understand this question. Finally, peers maintain contact with participants for the initial 90 days and use harm-reduction principles compared with the onetime interaction with social workers. It is therefore possible that there is a difference in treatment engagement beyond 30 days or an effect on recurrent overdose. Data collection addressing these secondary end points is in progress.

Given the high rates of treatment engagement after the ED visit, our data suggest that a behavioral intervention should be incorporated into routine ED care. Given the amount of time our interventionists spent at the bedside, a dedicated individual is likely needed to do this (beyond ED clinicians and nurses). In some health systems, this may be clinical social work staff, but in some EDs, peers or community health workers might be a more feasible resource.

### Strengths and Limitations

A few limitations should be noted when this study’s findings are interpreted. First, research assistants had contact with the participants in both groups. Although research staff were instructed and trained not to be involved in the intervention, it is possible that their interactions during consent or even during the self-administered assessments had an influence on participants, potentially making the groups more similar and thus biasing results toward the null. Second, as a pragmatic trial, we did not control or assess the fidelity of the intervention. Although our results reflect actual outcomes, it is possible that the interventions would have performed differently if they had been tightly controlled. Third, our study outcomes were captured with administrative data. Although this is a key strength of our study design, it is possible that participants could receive treatment that would not be captured by our data (eg, out-of-state or private programs not licensed by Department of Behavioral Healthcare, Developmental Disabilities and Hospitals). Although we do not believe it to be the case, if differential misclassification occurred, results would be further biased toward the null. Recovery from SUD has been described as a nonlinear process that often involves multiple recovery attempts.^34^ Participants may have varied in the number of recovery attempts, with some participants being further along in their process and more willing to enter into SUD treatment. This was not captured in our study and could lead to residual confounding. Fourth, our study lacked a true control group. For a variety of reasons, including standards of care in the state where this study was conducted, it was thought that randomly assigning patients to a sham intervention was unethical. Unfortunately, we cannot conclude that the behavioral interventions tested are superior to no intervention. Historical data suggest that this is not the case, and future work might seek to resolve this through causal inference methods using already collected data.

## Conclusions

This study suggests that an ED-based behavioral intervention is likely effective in promoting treatment engagement but that the person delivering the intervention may have less influence regarding the likelihood of a patient’s entering treatment immediately after the ED visit. Work is still needed to determine whether a peer-led intervention affects outcomes beyond 30 days or reduces the number of recurrent overdoses. In the era of highly potent synthetic opioids, highly responsive (ie, consultations within 30 minutes) ED-based interventions using appropriately trained personnel who support patients in accessing SUD treatment are vital. The outcomes from this study support the broad implementation of these services, irrespective of who would be providing them, in areas with high opioid overdose burden.

## Notes

Supplement 1.
Trial Protocol and Statistical Analysis Plan

Supplement 2. **eTable 1.** Enrollment in Substance Use Disorder Treatment Within 30 Days of Enrollment in the Navigator Trial **eTable 2.** Enrollment in Substance Use Disorder Program Within 30 Days of Enrollment in the Navigator Trial, Stratified by Reason for Emergency Department Visit and Treatment History

Supplement 3.
Data Sharing Statement
