# Supplementary material for: Effect of a Peer-Led Behavioral Intervention for Emergency Department Patients at High Risk of Fatal Opioid Overdose: A Randomized Clinical Trial
Source: JAMA Netw Open. 2022 Aug 9;5(8):e2225582. doi: 10.1001/jamanetworkopen.2022.25582 (PMC9364125; doi:10.1001/jamanetworkopen.2022.25582)
Supplement: Supplement 1. — Trial Protocol and Statistical Analysis Plan [file jamanetwopen-e2225582-s001.pdf]

SPECIFIC AIMS

Opioid overdoses are a leading cause of death for Americans under 50 years old, with recent years recording the most opioid overdose deaths on record.[1,2] US Emergency Departments (EDs) have seen a parallel increase in opioid-related visits (a 100% increase from 2005 – 2014).[3] ED patients presenting for an overdose are at greatly elevated risk for a repeat overdose and death.[4-6] **Thus, an overdose-related ED visit is both a critical and opportune time to prevent recurrent opioid overdose and overdose death through increased uptake in addiction treatment.**[7] The most effective means to promote engagement in treatment following an ED visit for opioid overdose remains unknown. To address this critical evidence gap, we will compare the effectiveness of two ED-based behavioral interventions to increase treatment uptake and reduce the risk of future overdose among ED patients who are at greatest risk of accidental drug-related death.

Rhode Island (RI) has the fifth highest rate of overdose mortality in the nation.[2] In response to RI's overdose crisis, in 2014, the state's largest ED (Rhode Island Hospital) began a proactive campaign to improve the care of overdose patients. The program includes an ED-based behavioral intervention either by in-house clinical social work staff or peer recovery support specialists ("peer navigators").[8] Following the introduction of these interventions in the ED, there was 10-fold increase in the proportion of patients engaging in addiction treatment within 30 days of the initial ED visit. While this preliminary data is promising, the effectiveness of the peer navigators versus the social work intervention is not known. Both interventions are intended to promote early treatment engagement after the ED visit, but peer navigators are distinguished from hospital-based staff in that they: (1) help navigate personal and structural barriers to treatment utilizing both training and real-life experience (e.g., overcoming stigma, transportation barriers, relapse triggers); (2) continue to engage patients beyond their ED visit (when they are especially vulnerable to recurrent overdose and death); and (3), promote long-term retention in treatment programs, including medication assisted treatment (MAT), through a long-term relationship that was established at the ED visit. Although peer support is a recognized component of many successful addiction treatment and recovery approaches,[9-15] the use of peer navigators in the ED setting to care for overdose patients is highly novel.

The Rhode Island peer navigator intervention model is being expanded nationwide, yet the effectiveness of this approach, relative to standard interventions delivered by hospital staff, is unknown. The ED-based peer navigator program was developed in alignment with the federally funded Rhode Island State Targeted Response initiative and is a key component of Rhode Island's strategic action plan to address opioid addiction and overdose.[16] Of critical significance to this proposal, there is substantial interest from other states in replicating RI's peer navigation model.[17] In light of this growing national attention, a recently published J-PAL policy brief made a call for formal evaluations of ED-based peer interventions.[18]

To address this urgent need, we will conduct the Navigator Trial, a randomized controlled trial (RCT) of early ED behavioral interventions following an opioid overdose. **We hypothesize that peer navigation will result in greater early treatment engagement and reduction in recurrent opioid overdose compared to a standard intervention delivered by a clinical social worker.** A key strength of this study is the ability to link patient data with statewide administrative databases to ascertain objective outcome data on all study participants. These databases are part of the state's robust CDC-funded overdose surveillance platform, www.PreventOverdoseRI.org, and include information on all overdose events, as well as admissions and discharges to all licensed substance abuse treatment facilities in Rhode Island.

Primary Aim 1a. We will determine the effectiveness of peer navigation versus a standard behavioral intervention delivered in the ED to overdose patients and those at risk of recurrent opioid overdose. 650 patients will be recruited between Rhode Island Hospital (RIH) and The

Miriam Hospital (TMH) ED (n=325 per arm) and followed for 18 months. Effectiveness will be measured objectively through linkage to administrative statewide databases, with two primary endpoints: (1) engagement in formal addiction treatment (e.g., inpatient services, outpatient services, MAT) from a licensed substance abuse treatment provider within 30 days following the ED visit, and (2) reduction in 18-month recurrent ED visits for an opioid overdose. Exploratory outcomes of interest are: overdose fatality, repeat ED visits related to opioids, and successful completion of an addiction treatment program and/or long-term retention in MAT.

Primary Aim 1b. We will assess the impact of two interventions from the RCT (peer navigation OR social worker intervention n=650) against a "no treatment" control group (n=325) on the outcomes in 1a. Opioid overdose patients who decline treatment as part of the RCT interventions, yet consent to follow-up, ongoing record review, and administrative database linkage will be utilized as controls.

A total of 975 (325 receiving Peer Navigator services, 325 receiving Social Work services, and 325 who declined receiving either of these services) subjects will be enrolled in this study.

Secondary Aim. We will explore if there is heterogeneity of treatment effect related to patient characteristics. We anticipate that there will be individuals within each treatment arm who will vary in their response to the intervention. Specifically, we will examine if the effects of the interventions are modified by baseline characteristics such as age, sex, race, type of opioid used, and history of comorbid chronic pain, depression or PTSD. Understanding these factors will allow us to further optimize subsequent interventions.

In sum, this study will evaluate a government-funded program that is being successfully delivered under real-world conditions, but that has not been rigorously tested. This trial will provide critical data regarding the effectiveness of ED-based peer navigation services, with major implications for Rhode Island and other states.

Qualitative Aim. From the participants recruited, a subset of 30 patients will be interviewed 2-10 days following their enrollment to better understand the reasons why they may have refused or accepted Social Work or Peer Navigator services, their experiences with these services, and provide a foundation for improving such services.

RESEARCH STRATEGY

A. Significance

A.1. Opioid overdose prevention is urgently needed. In 2015, over 33,000 people died from opioid-related drug overdoses in the United States, more than any previous year on record.[1,2,19] The opioid epidemic has not abated despite a recent overall decrease in the number of opioid analgesics prescribed by US providers.[2,20-23] While opioid analgesic prescribing is believed to have initially driven this opioid crisis,[24] the epidemic has rapidly evolved. Heroin use is increasing for the first time in more than a decade,[19,25-30] and overdose deaths due to potent synthetic opioids, such as fentanyl and carfentanil, are on the rise.[31-37] In light of this public health crisis, there is a pressing need for novel approaches to reduce overdose deaths and mitigate other opioid-related harms. **In response to this epidemic, the primary goal of our study is to intervene on a subset of persons who are among those at highest risk for drug-related mortality — patients presenting to an emergency department (ED) for an opioid overdose** and those at risk of recurrent opioid overdose**.**

A.2. The Emergency Department (ED) as a critical intervention site. Between 2005 and 2014, the national rate of opioid-related ED visits increased almost 100%.[38,39] Alarmingly, ED

visits for drug overdoses continue to rise.[3] Rhode Island ranks as the second highest in the nation for the rate of opioid-related ED visits.[38] Over the last 18-month period, over 2,200 opioid overdose patients have been treated in EDs across the state. In the year after presenting to the ED for an overdose, a person is a heightened risk for all-cause mortality (7-fold increase).[5] **The ED may be the only contact point with the health care system and is thus both a critical and timely place for intervention,** using the reason for the ED visit itself (i.e., overdose) as an opportunity to identify patients at highest risk of drug-related mortality and deliver behavioral interventions.[40-44]

A.3. Peer-based interventions are a proactive approach. The majority of overdose patients in the state present to the Rhode Island Hospital ED, one of the study sites for this investigation. Of those presenting to the RIH ED, nearly half receive some sort of behavioral intervention in the ED, approximately 40% of whom currently receive counseling from a peer navigator, see

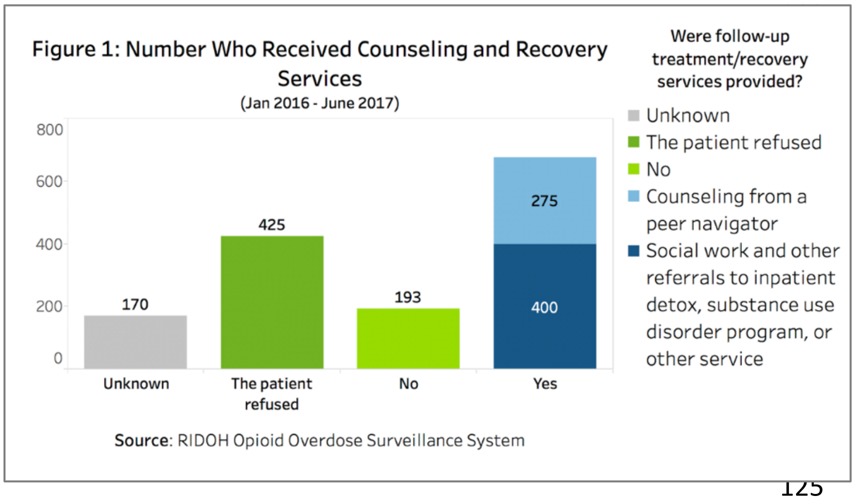

Figure 1: Number Who Received Counseling and Recovery Services (Jan 2016 - June 2017)

Figure 1. Peer navigators are individuals in long-term addiction recovery uniquely positioned to form supportive relationships with those struggling with addiction, helping patients "navigate" obstacles to recovery through problem solving, goal setting, avoiding relapse triggers, and planning or obtaining services. Continued contact with the patient after the ED visit is a critical component of the peer-based model. Finally, peer navigation programs are distinct from other behavioral interventions in that they are centered on the recovery process, cultural diversity and inclusion, community participation, peers helping peers, and leadership development.[45] **Despite the promising approach, no studies have rigorously evaluated whether peer-based behavioral interventions result in improved outcomes for patients treated for an opioid overdose.**[18]

Peer-based interventions have been shown to be an effective component of care across non-clinical settings,[46] and in other aspects of health care for other conditions.[47-50] There are also early examples of peer-directed interventions in the ED for other conditions, but not opioid overdose.[51,52] **This trial is the first to test whether a peer-based model is more effective than a brief intervention delivered by clinical social workers for patients at risk of subsequent overdose**.

A.4 Rapid dissemination of study findings and integration with key public health stakeholders. Several factors ensure that the results of this study will inform future care for overdose patients in Rhode Island and beyond. First, strong collaborations with state agencies will support expansion of peer-based interventions if they are found to be effective. As demonstrated by our letters of support, our team has fostered a strong and established network of organizations, public health experts, and political leaders working collectively to reduce opioid overdose deaths across Rhode Island. **This strong foundation will allow dissemination of project results to key stakeholders, ensuring that knowledge gained from this study positively impacts care for overdose patients in the state.** Second, the Rhode Island Department of Behavioral Healthcare, Developmental Disabilities, and Hospitals (BHDDH), which funds the peer recovery program through a SAMSHA initiative, has plans to increase the

number of certified peer navigators by 50% starting in 2018. The Director of BHDDH, Rebecca Boss, has affirmed that “if the proposed trial demonstrates peer-based ED navigation to be superior to current standards of care for patients admitted to the ED for an overdose, we would continue to expand this program throughout hospitals across Rhode Island” (see letter of support). Finally, we will work with the Laura and John Arnold Foundation and other partners to broadly disseminate our finding to other states, many of which have already adapted the Rhode Island peer recovery model.[53]

B. Innovation

B.1 Rhode Island is a national leader in developing highly innovative programs for overdose prevention. The state has established national models for overdose prevention and intervention, and has encouraged community-level innovation. In response to the growing overdose crisis and with funding from BHDDH, in 2014, the community-based organization Anchor Recovery Community Center launched a new initiative, AnchorED. Anchor Recovery Community Center (ARCC) is Rhode Island’s first and only community organization run by and dedicated to those living in recovery from substance use disorders. Focusing on a peer-to-peer support model, ARCC helps those living with substance use disorders maintain long-term recovery, improve social connectedness, improve self-sufficiency, and live healthier lives. The AnchorED program takes the peer model into the ED, dispatching trained and certified peer navigators to EDs throughout RI to provide recovery support and services navigation to individuals who have experienced an opioid overdose (see additional details in section C.3). **Importantly, AnchorED is the first program of its kind in the nation**.[54] Despite a paucity of evidence to demonstrate effectiveness and improved long-term outcomes for overdose survivors, jurisdictions in New York, Connecticut, Massachusetts, New Hampshire, and New Jersey are in the process of creating programs based on the Rhode Island peer model.[55] **Given the intense interest in peer recovery models in settings across the nation, the results of our study have a strong potential to fundamentally shift clinical practice paradigms for treating overdose patients in the ED.**

B.2 A data-rich policy and public health environment to boost study rigor. In 2015, Governor Gina Raimondo signed an executive order to establish the Rhode Island Overdose Prevention and Intervention Task Force. This task force charged experts (including Dr. Marshall, Co-PI) to develop a strategic plan that would guide efforts to tackle the state’s overdose crisis.[56] An important outcome of this plan was a comprehensive data-sharing framework between key state agencies and academic researchers.[57] This data sharing agreement offers unprecedented access to population-based overdose morbidity and mortality data. As a result, our team has the capacity to link Navigator Trial patient data with multiple statewide administrative databases (e.g., medical examiner case files, behavioral health data on treatment admissions) to ascertain objective outcome data. Importantly, by linking patient data to these datasets, we will be able to assess objective primary and exploratory outcomes (e.g., engagement in treatment, overdose death) for all study participants. **The ability to conduct robust data linkages is an important methodological innovation of our proposed project,** and one that overcomes the limitations frequently present in other observational and experimental studies of overdose patients (e.g., biases related to self-reported outcomes and loss to follow-up). Finally, management of these datasets and support for the state’s electronic overdose surveillance system (http://www.PreventOverdoseRI.org) are funded by the CDC; thus, the proposed project leverages existing infrastructure and administrative data to reduce overall study costs.

C. Approach

C.1. Study overview, setting, and target population. We will compare the effectiveness of peer navigation versus a standard social work intervention delivered in the RIH and TMH EDs following an ED visit for opioid overdose. 650 patients treated for an opioid overdose or at risk

for an opioid overdose (defined below) will be randomized to receive either Social Work or Peer Navigator services (n=325 per arm) and followed using administrative datasets. Our primary outcomes will be: (1) engagement in treatment within 30-days after the ED visit, and (2) recurrent ED visit(s) for opioid overdose over the 18-month follow-up period. Additionally, we will also enroll a "no intervention" self-selected control group (n=325) at both hospitals to assess the impact of the two interventions from the RCT (peer navigation OR social worker intervention) against no intervention. A total of 975 (325 receiving Peer Navigator services, 325 receiving Social Work services, and 325 who declined receiving either of these services) subjects will be enrolled in this study. We decided to allow patients to self-select into the "no intervention" control group. Given that an offer of one of the two behavioral interventions has become the standard of care for overdose visits in our ED, we believe that it would be unethical to randomly assign individuals to a "no intervention" control condition. We considered using another RI-based ED as the control condition, but other EDs across RI have already adopted our program of offering behavioral intervention following overdose. We will use quasi-experimental statistical analyses to control for selection bias and confounding that could arise from non-random assignment of the controls. The two sites where this investigation will take place are Rhode Island Hospital and The Miriam Hospital ED in Providence (RIH being Dr. Beaudoin's primary practice site). RIH is an academic, tertiary care, level one trauma center with >105,000 annual ED visits, that serves a heterogeneous and demographically diverse patient population. The RIH ED cares for the majority of opioid overdoses throughout Rhode Island. In 2017, RIH has recorded an average of 85 opioid overdoses per month (~1,000/year). As such, RIH is an ideal site for the proposed study.

We will recruit adult ED patients in both hospitals who are: (1) being treated for an opioid overdose, <u>or</u> (2) have had an opioid overdose in the past 12-months (identified by self-report during screening or in review of the EMR); <u>or</u> (3) are presenting with a visit related to illicit injection opioid use (e.g., cutaneous injection-related infection, opioid withdrawal, endocarditis). We are specifically targeting patients with a current or recent opioid overdose and those who inject opioids illicitly, as they are at highest risk for opioid overdose and death.[4,58]

C.2. A highly experienced research team will ensure this project's success. This proposal leverages thousands of hours of work by the investigative team in conducting clinical trials and observational studies (see biosketches). For example, co-PI Dr. Beaudoin has an outstanding track record of leading studies that recruit patients in a busy ED environment, overseeing screening evaluations and assessments for thousands of patients, and successfully conducting follow-up in a challenging population of patients with addiction. Dr. Marshall is a nationally recognized expert in substance use epidemiology and, as the director of the state's overdose surveillance system, www.PreventOverdoseRI.org, brings a wealth of experience in the management and analysis of overdose-related patient data. In sum, Drs. Marshall and Beaudoin have the complementary expertise necessary to achieve the aims of this project and are surrounded by a team of multi-disciplinary co-investigators and consultants with expertise in addiction, behavioral psychology, and peer navigation

C.3. Overview of behavioral interventions available at the Rhode Island Hospital and The Miriam Hospital ED. Currently, standard of care is to receive a behavioral intervention, *either* peer navigation or social work. Whether a patient sees a social worker or peer navigator varies and can depend on the time of day, availability, and preferences or biases of the provider or patient. Once funded, we have permission and explicit support from the peer and social work programs to randomize patients to one of the two interventions (see letters of support).

<u>Peer Navigators:</u> The Peer Navigators of the Anchor Recovery Community Center (ARCC) of Rhode Island will deliver the peer navigation arm of the intervention. They are available 24 hours a day, 7 days a week to provide recovery support, referrals, and ongoing engagement for

ED patients after discharge. They arrive in the ED within 30 minutes of consultation, assess individuals for readiness to seek treatment, provide linkage to treatment, and educate on overdose prevention and response, including naloxone administration. Following the ED visit, peer navigators follow up with patients within 24-48 hours and maintain contact and services navigation for at least 90 days following ED discharge, on a weekly basis and more frequently as needed.

To become a peer navigator, applicants must be in long-term recovery (≥2 years) and undergo a rigorous training program. The peer navigators learn motivational interviewing techniques and the transtheoretical model of behavior, also known as the "stages of change".[59] Motivational interviewing is considered to be a patient-centered, non-judgmental approach that aims to enhance the intervention recipient's intrinsic motivation to change and building on their self-identified goals and strengths.[60-62] The "stages of change" framework is widely applied to substance use disorders, and proposes that individuals move through discrete steps when making behavior change: Pre-contemplation, Contemplation, Preparation, Action, and Maintenance.[59,63] In addition to this standard framework, peer navigators support individuals' self efficacy and prevent relapse by addressing social, environmental, and personal factors, such as awareness and avoidance of relapse triggers, poly-substance use, stigma of addiction/treatment, knowledge of treatment services, and financial or transportation barriers to treatment. **They are uniquely poised to deliver this portion of the intervention by coupling training with real-life experience in way that cannot be mirrored by hospital-based staff.**

<u>Licensed Clinical Social Workers</u>: The staff social workers of the Department of Social Work at RIH and TMH will deliver the social work arm of the intervention. There are 35 full and part-time masters-level social workers, all licensed by the state at Rhode Island Hospital and 3 full-time masters-level social workers at The Miriam Hospital . A social worker is available 24 hours a day, 7 days a week to see consultations in the RIH ED while at The Miriam Hospital, they are available from 7 AM-11 PM, 7 days a week. They generally respond to consultations within 30 minutes. They are currently trained and available to deliver interventions to ED patients with OUD. Clinical social workers are capable of delivering a variety of interviewing and intervention techniques that are rooted in models of social work theory and practice models.[64-68] Social work practice models are strategies that the social worker can incorporate into their interventions in order to help people meet their goals (e.g., task-centered practice,[69] cognitive behavioral therapy,[70] the crisis intervention model[71]). For the purposes of the study, social workers will also receive a refresher course in motivational interviewing and a stages of change framework as described above. Although this intervention and theoretical framework is similar in some respects to the peer navigation arm, the social work intervention is a single brief intervention with referral to treatment that may also rely on social work theory and practice. The peer navigation arm is distinguished by many unique aspects as outlined in sections above, and highlights that the person delivering the intervention may be as or more important than the intervention itself.

C.4. Participant eligibility criteria, recruitment, and enrollment. A consecutive sample of ED patients presenting to the RIH and TMH EDs will be assessed for eligibility. The RIH ED has 11 full-time research assistants (RAs) available to recruit 24 hours per day, 7-days per week. At TMH ED, there are 3 full-time RAs available to recruit participants from 7 AM-11 PM, 7 days a week. Participants at TMH ED will be recruited during the hours in which social workers and RAs are available as described above. Participants will be identified by screening the ED's electronic medical records (EMR) or by referrals from ED treating providers. Patients who meet the initial eligibility screen will undergo a further in-person assessment by a study RA. Participants will be eligible if they are: (1) English speaking, (2) 18 years of age or older, and (3) are being treated for an opioid overdose *or* identified as having had an opioid overdose in the past 12 months *or* are being treated for a visit related to illicit opioid use (e.g., abscess, opioid

withdrawal), (4) are identified as having an alcohol OR other drug use disorder (excluding marijuana) PLUS illicit opioid use in the past 6 months, (5) and are able to provide informed consent. Participants are ineligible if they are critically ill or injured, are previously enrolled in the trial, in police custody or incarcerated, pregnant, or live outside of Rhode Island or Southeastern Massachusetts where they primarily receive their care (patients must live in state OR within Southeastern Massachusetts but primarily receive care in Rhode Island to link to administrative database). Patients who are critically ill will be eligible once cleared by their physician.

C.4.1. After screening, if the patient is eligible and willing to participate, then full written informed consent will be obtained. After obtaining consent, the RA will randomly assign that patient (1:1 allocation) to the peer navigation arm or the social work arm using sealed envelopes. The randomization schedule will consist of permuted block sizes stratified on gender and age. Both gender and age may be important determinants of the effectiveness of treatment, and will be examined as moderators in our exploratory analysis. This schedule will be maintained by the study analyst; neither the study PIs, nor the recruiting staff will be aware of the schedule. We anticipate the intervention will begin within 30 minutes of randomization.

C.4.2. Patients who decline to be randomly assigned to one of the two treatment groups as part of the study are also eligible to be part of the “no intervention” control group. Such patients will be offered the opportunity to be assessed by either a peer navigator, social worker, or both. If they decline to be assessed by either a peer navigator or a social worker, they will be eligible to be in the control group. Eligible control patients will be asked for their permission to participate in follow-up assessments and other data collection procedures throughout the study period.

C.4.3. Study assessments. Baseline assessments in the EDs will collect information about socio-demographic characteristics, medical history, medication use, substance use, prior addiction treatment, pain symptoms, and depression. The survey forms may be RA or self-administered. Sections on more sensitive topics will be self-administered unless the participant requests that the research assistant administer it. Patients in the randomized group will receive a $40 gift card on the day of enrollment for completing the baseline survey while those in the control group will receive a $25 gift card for completing their baseline survey. See appendix for instruments.
Qualitative interviews will be conducted by telephone or in-person 2-10 days following enrollment in the ED. These audio-recorded interviews will take about 45 minutes to an hour to complete and participants will receive a $50 gift card for their time. In-person interviews will be held at a Lifespan facility or a public community space that has been agreed upon by RA(s) and participant. Transportation in the form of cab voucher and RIPTA bus-pass will be provided to those requiring it.

C.4.4. Qualitative-Interview Procedures: The protocol will apply the same recruitment methodology as the Navigator study and patients will be approached after they have completed their assigned Navigator treatment arm, or after assessments for the control group. Navigator participants will be eligible for participation in the interviews if they have completed the baseline assessment and have been assigned to one of the intervention arms, and received the intervention in the ED, or control arms of the study, have a working telephone number or are prepared to return a Lifespan facility to participate in the patient interview protocol. Qualitative interview audio recordings will be transcribed by a HIPAA compliant transcription service.

C.4.6 Qualitative Data Analysis

After the audio-recordings have been transcribed and cleaned by the RA, the investigators will to read the transcripts and meet on several occasion to conduct an iterative content analysis, based on the approach of the immersion-crystallization method of qualitative analysis. This qualitative approach involves individual followed by a larger group determination of emerging themes around the content of the interviews discussing the data as a group to determine emerging themes, salient across all groups, and also those themes reflected by the subgroups sampled. From this thematic analysis a code book will be developed and NVivo qualitative data coding software will be employed to manage and sort the data. Coding discrepancies emerging during investigators meeting will be discussed and resolved; to collectively determine the final code. Following NVivo analysis the investigators will meet to complete the interpretation of the themes.

C.5. Definition and operationalization of primary outcomes. This RCT will have two primary endpoints: (1) 30-day treatment engagement, and (2) recurrent ED visits for an overdose. We will obtain objective assessments of these outcomes through the use of statewide administrative database as outlined below:

30-day treatment engagement (endpoint 1). The first primary outcome, engagement in addiction treatment, will be defined as the proportion who are admitted to a formal addiction treatment program within thirty days following the initial ED visit. This outcome was chosen because a key short-term goal of the ED behavioral intervention is to promote early treatment engagement. This outcome will be assessed using BHDDH and Prescription Drug Monitoring Program (PDMP) records. The BHDDH database contains information on all admissions to publicly funded substance abuse treatment programs in the state. We will define treatment engagement as admission to any of the program types licensed by BHDDH, including inpatient detoxification, day treatment programs, residential treatment, intensive outpatient services, and opioid treatment programs (i.e., methadone). Second, we will query the participant's RI PDMP records in order to identify enrollment in office-based buprenorphine therapy. The RI PDMP manages a database that contains information on all prescriptions for schedule II-IV substances filled in the state. The database is updated daily; all pharmacies are required to report prescriptions within 48-hours of the fill date. The database includes information on the patient (e.g., name, sex, birth date, address including zip code), the prescription filled (e.g., quantity, days supply, national drug code number), and prescriber/pharmacy data. Pharmacies are required by law to report prescriptions to the PDMP regardless of payment type. All records will be linked deterministically to participant data using identifiable information (e.g., name, social security number) within the Stronghold computing environment, a HIPAA-compliant server maintained by Dr. Marshall's team at Brown University. Our research team has experience extracting and analyzing PMDP and BHDDH data for statewide surveillance purposes.[72]

Recurrent ED visits for overdose (endpoint 2). The second primary outcome, recurrent ED visit for overdose, will be defined as the proportion of participants who are treated in any Rhode Island ED for an opioid overdose at any time during the 18-month follow-up period following the initial ED visit. Recurrent ED visits for overdose were chosen as the second primary outcome as a long-term goal of the ED behavioral interventions is reduce fatal and non-fatal overdose. Two data sources will be used to assess this outcome. First, we will access the electronic medical records (EMRs) of the 12 EDs in Rhode Island through the Rhode Island Quality Institute Statewide Health Information Exchange. This data source will be made accessible through Brown's Advance-CTR Unified Research Data Sharing Access (URSA) infrastructure. This unified data system provides access to EMR data from all major health systems in Rhode Island. Thus, we will capture repeat visits for an opioid overdose that occur in all 12 EDs in Rhode Island. We will define an ED visit for an opioid overdose based on CDC guidelines for all

opioid poisonings (which includes illicit opioids) and utilizes International Classification of Disease (ICD) coding.[73] Second, we will query the RI Department of Health (RIDOH) Opioid 48-Hour Overdose Surveillance System. The RIDOH mandates all suspected opioid overdose cases presenting to an RI hospital be reported to the department within 48 hours.[74] This data source will capture recurrent overdoses not identified by ICD codes in the unified EMR data system, and also contains additional fields of interest (e.g., pre-existing risk factors for overdose).

C.6. Statistical analyses. For all analyses, routine procedures will first be conducted to ensure data accuracy/adequacy. We will use an intention-to-treat (ITT) approach in all analyses to address potential problems inherent in following only intervention completers; a sensitivity analysis ("per protocol") will be conducted among only those that complete the ED intervention. Prior to examining intervention effects, we will first assess the success of randomization on pre-intervention characteristics using analyses of variance (ANOVA), with intervention group as the predictor variable. If there are differences in baseline characteristics, we will include these covariates in the primary outcome analyses, as described below. Given use of administrative data sources we anticipate minimal missingness in our final dataset. However, missing covariate data will be handled using multiple imputation performed in two stages using chained equations that specify the conditional models for all of the variables with missing values.[75-77]

C.6.1. Effectiveness, 30-day engagement in treatment (primary endpoint 1a). We will compare the effectiveness of the peer navigation versus social work intervention on increasing engagement in formal addiction treatment within 30 days of the initial ED visit. As the primary analysis, we will compare the proportion who are admitted to a licensed addiction treatment program (using chi-square analysis) between the two groups. Next, logistic regression models will be used to determine the independent effect of the intervention arm on 30-day treatment admission, adjusting for any baseline covariates as described above.

C.6.2 Effectiveness, any behavioral intervention versus "no intervention" treatment (primary endpoint 1b). We will conduct analyses similar to those described above to determine whether participants who receive any intervention have improved outcomes compared to those who refuse to receive any behavioral intervention. The primary independent variable of interest for Aim 1b is treatment versus control group membership. However, unlike Aim 1a (in which two randomized interventions are compared), patients non-random assignment (self-selection) into the control group. As such, we will need to account for factors that may be associated with refusing a behavioral intervention in the ED. We will use inverse probability of treatment weight (IPTW) techniques to account for: (1) baseline risk factors that predict control group membership, and (2) post-randomization confounding.

C.6.3. Effectiveness, recurrent ED visit for an opioid overdose (primary endpoint 2). We will compare the effectiveness of the peer navigation versus the social work intervention on preventing subsequent ED visits for opioid overdose. As the primary analysis, we will compare the overall proportion of patients experiencing a subsequent opioid overdose over the 18-month follow-up period between the intervention groups (using chi-square analysis). Next, logistic regression models will be used to determine the independent effect of the intervention arm on recurrent ED visits for opioid overdoses, adjusting for baseline covariates.

In **exploratory analyses**, we will also examine a number of other outcomes, including: overdose *rates*, overdose death (all overdose deaths in RI are analyzed by Dr. Marshall's team and can be linked deterministically to patient data), and successful completion or retention in addiction treatment. Successful completion and/or retention in addiction treatment will be defined based on discharge data collected in BHOLD and prescription refill data in the PDMP

(e.g., on MAT for ≥6 months). We will examine the time to ED visit for an opioid overdose using a Kaplan-Meier analysis. Patients will be censored at the end of the 18-month follow-up period, considered the last point of contact. We will use Breslow's method to test if the time to subsequent opioid overdose rates differs between the groups. Next, Cox proportional hazards modeling will be used to estimate hazard ratios (HRs) and corresponding 95% confidence intervals (CIs) for occurrence of repeat overdose between groups. HRs will be adjusted for clinical and demographic characteristics believed to predict the outcome of opioid overdose in order to adjust for possible residual confounding and treatment-factor interactions. Finally, since participants may experience multiple opioid overdoses during follow-up, we will also conduct recurrent-event survival analyses.[78] These models extend the Cox model approach and allow for estimation of hazard ratios pooled across repeated periods at risk.[79] Finally, we will examine if heterogeneity of intervention effect is modified by age, sex, race, pre-existing chronic pain, past treatment history, and reason for presentation to the ED. We will perform stratified subgroup analyses to determine if treatment effects vary between groups of individuals.

C.7. Feasibility and Sample Size Calculation. First, we assume that ~2,000 patients will be treated at the RIH or TMH ED for an opioid overdose over the 24-month recruitment period; this is based on data from the RI Opioid Overdose Surveillance System and is a conservative estimate that does not include other eligibility criteria (e.g., recent overdose). Next, we assume that 65% (n=1,300) of these patients will be willing to be screened (prior studies of behavioral interventions for drug use at the RIH ED have had screening rates > 80%).[80] Third, we assume that ~50% (n=650) of these patients will be eligible and randomized to an intervention arm (n=325 per arm). Thus, we estimate that 24 months will be required to recruit 650 participants. Given our use of objective outcome data from administrative datasets, we do not expect dropout to significantly impact our statistical power, but our power calculations conservatively reflect a 10% loss to follow-up rate. Will attempt to also enroll similar numbers in the control arm (n=325).

For our <u>sample size calculation</u>, we assumed that 7% of participants in the social work arm will enroll in a formal treatment program within 30 days of ED discharge (based on preliminary data from the state and RIH). Given this, we have >80% power to detect a two-fold increase (i.e., >7% absolute increase) in the rate of 30-day treatment engagement between the two arms (Figure 2A); this increase has been deemed a bench-mark by key state stakeholders. For primary endpoint 2 (recurrent ED visit for an opioid overdose), we assumed that 15% of patients in the social work arm will have a recurrent ED visit for an opioid overdose within 18-months of their first visit. This estimate is based on a chart review of 374 patients after program implementation of the RIH ED behavioral intervention program and in recently published data by Banta-Green et al.[81] The latter study from Washington State found around a 20% incidence of overdose within 18-months following of an initial ED visit. Our assumption of a 15% incidence of repeat ED visit for overdose is conservative compared to this finding, particularly in light of the fact the Rhode Island has nearly twice as many overdose deaths per capita than Washington State.[82] We will have >80% power to detect a 50% relative reduction (7.5% absolute reduction) in the risk of recurrent overdose within 18 months of their ED visit (Figure 2B), this reduction was felt to be clinically relevant and commensurate with statewide goals in reducing overdose via various strategies.

Figure 2A: Power for Endpoint, 30-day treatment engagement

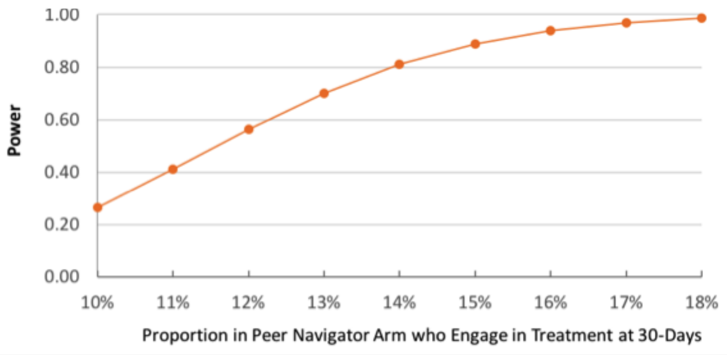

<u>Design consideration, length of follow-up</u>: We chose to evaluate outcome date within the first 18 months after the initial ED visit for two main reasons. First, the risk of recurrent overdose appears to level-off by about 18 months, meaning that most individuals who will

experience another overdose will do so within the first 18 months.[81] Second, given the urgent need to have an evidence-based evaluation of peer-led behavioral interventions for OUDs, a shorter length of follow-up would allow us to disseminate our study findings sooner. However, we recognize that there is potential value in having a longer follow-up period both from an impact standpoint and also in terms of statistical power to detect a difference between the treatment groups. Figure 2b compares the difference in power between 18 and 30 month study endpoints (> 80% to detect a RR=0.5 for 18 months and RR=0.58 for 30 months; absolute rate of overdose in the intervention arm of ~7.5% at 18 months and ~11.5% at 30 months, note that this assumes an 20% incidence of overdose at 30 months). Therefore, as a contingency plan, we could extend the length of follow-up by one year (30 months total) based on 18 month outcome analyses. This is possible because of the use of administrative data for the outcomes assessment, but would require additional cost (approximately $120,000). We propose to make this decision in conjunction with the LJA Foundation should this proposal be approved for funding.

Figure 2B: Power for Endpoint 2, Recurrent ED visit for overdose

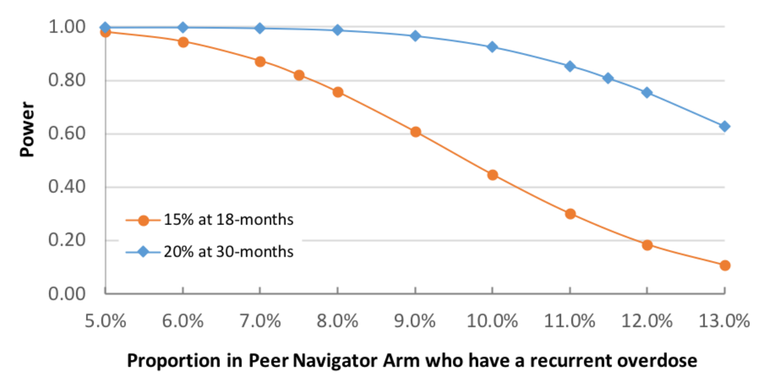

Appendix 1: **Protocol for Navigator Patient Interviews**

For use in both Rhode Island Hospital and The Miriam Hospital ED study sites.

Purpose: The purpose of the patient interviews is to understand the experiences of ED patients who have agreed to participate in the study after meeting study eligibility criteria. These will be semi-structured interviews to allow for patient directed reflections on the interactions they experienced as a patient before they were recruited into the study, the decision making that led them to agree to participate in the study, their experience with the peer navigator or social worker they were randomized to be

exposed to, and for those patients in the control group (declining either social worker or peer navigator), their reasons for not wanting to see either treatment option. The key research questions to be addressed in the qualitative portion of the navigator study are around the ED research experience , how this could be improved, and the effect that the Navigator study has on their motivation to engage in treatment, in the ED and after discharge, for their opioid use. Below are specific themes that the patient interview protocol will address:

1. What influences the patient to be part of the Navigator study- were their reasons apart from compensation?
2. What was their feedback about the Navigator process: assessment and randomization, follow up schedule and assessments? What could have been done better or differently?
3. How was the transition from the assessment to engaging with the peer navigator or social work handled (e.g. explanation of who they would be talking to and what that interaction would be like; length of time waiting for interaction)?
4. Before randomization, did the patients have a preference about talking to a social worker or peer navigator? What were the expectations about the intervention before it took place, and were there expectations met or altered after the intervention?
5. Had the patient, outside of the ED, talked to a social worker, peer navigator, or other resource about their opioid use? If yes, how did the experience in the ED patients compare to prior talks/counseling on their opioid use?
6. Did the patient engage with the peer navigator outside of the ED? If yes, why? If no, why? For patients assigned to the social work intervention, would it have been helpful to transition them into community treatment to have continued contact with the social worker after being discharged from the ED?
7. For all patients, at the time of being recruited into the Navigator study were they currently receiving any active treatment for their opioid use? If yes, what was it and how did this influence their decision to take part or not in the Navigator study?
8. For all patients, prior to being approached to take part in the study had any non-research ED staff (physicians, nurse practitioners, nurses, EMT) talk to them about their opioid use and suggest that they seek treatment? If yes, who was it and what were their responses to this suggestion?
9. For patients in the control arm, why did they refuse to talk to a peer navigator or social worker?
10. What are patients’ expectations and tolerance for treatment for their opioid use as part of their ED visit (e.g. MAT) and after discharge (e.g. community treatment)?
11. If the patient was being treated for an OD, were they offered naloxone, how was that experienced by the patient ,and what was the patient’s decision making process in accepting or not accepting naloxone?
12. What influenced the patient’s decision to engage, or not, in treatment for their opioid use?

13. For patients treated for an unintentional opioid overdose, if they were offered naloxone, did they accept or refuse, and what influenced that decision
14. For all patients, what was their overall ED treatment experience (for the patient's presenting complaint) and how did this influence them in their decision to take part in the study, and to agree or not to talk to a peer navigator or social worker?
15. Did the patient perceive stigma at any point during their ED visit or as part of the Navigator study?

Screening and recruitment. The protocol will apply the same recruitment methodology as the Navigator study and patients will be approached after they have completed their assigned Navigator treatment arm, or after assessments for the control group. Navigator participants will be eligible for participation in the interviews if they have completed the baseline assessment and have been assigned to one of the intervention arms, and received the intervention in the ED, or control arms of the study, have a working telephone number or are prepared to return to a LifeSpan affiliated facility or public community space to participate in the patient interview protocol. We will use a selective sampling approach, where the RA will approach patients eligible to participate in the interviews from the recruitment schema below. At the time of consent in the ED into the navigator study the patient will be asked to consent to possibly be contacted to take part in the qualitive interview components of the study. Within 2-10 days after the ED visit, which will be sufficiently recent to remember the ED visit, and, for the peer navigator group, sufficient time will have passed to ensure that there has been at least one contact with the per navigator.

The RA will call a selected participant and ask if they w oud like to participate in the semi-structured interview will be conducted by phone or in-person and will be audiotaped for transcription and later analysis. Verbal consent will be obtained from the participant. The RA will give the option of completing the interview by phone or in person at a Lifespan facility or a public community space that has been agreed upon by RA(s) and participant. If the participant agrees to a telephone interview the RA will remind the participant that the interview will be audio-recorded, switch on the audio recorder and proceed with the verbal consent statement (see Procedures below). Alternatively an appointment for the in-person interview will be scheduled and the same verbal consent procedure will be conducted..

Sample size for semi-structured interviews. Based on literature on usability data exploring consumer feedback on new designs, interviews with up to five participants from each group of users is sufficient to provide detailed insight into positive and negative experiences before saturation of feedback is reached. Below is the schema for recruitment from the relevant patient constituents who will be interviewed (n= 30 in total). The current proportion of 25% female recruitment will be reflected in the sample interviewed. The RA will continue recruiting participants until all required subgroup cell numbers have been obtained.

Navigator participants

N = 30

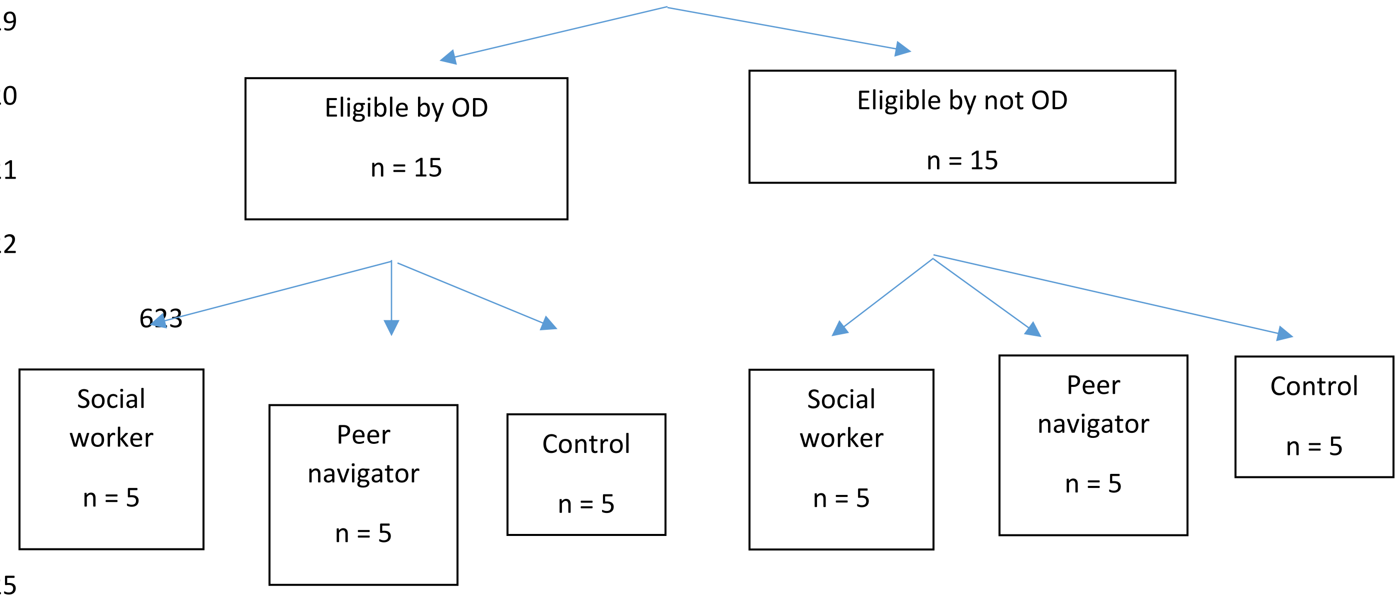

We anticipate hearing distinctly different reports of the overall ED treatment experience and reflections on the Navigator research and treatment experience in the three groups, regardless of eligibility criteria. We anticipate that the interviews will take between 45 minutes to an hour to complete, and to reflect this additional request on Navigator participants' time we will offer participants a $50 gift card for participation. Participants will be offered transportation to bring them to the interview if they chose the in-person option. The participant data will be confidential and will not be linked with any other research data they provide as part of the main Navigator research study.

Procedure. At the start of the telephone or in-person interview the person will be reminded that the interview will be audio recorded and asked to consent to this- this consent will be repeated when the audio-recorder is activated. The following preamble will be recited to the participant:

[TURN ON TAPE RECORDER; READ]:

PREAMBLE/CONSENT [READ ALOUD, CONSENT MUST BE DOCUMENTED BY RESEARCH ASSISTANT]: Hi, my name is **[interviewer name]** and I/m a member of the Navigator research team that will be conducting this interview with you today. We want to ask

you about your experiences of taking part in the Navigator study when you were treated in the emergency department at (*RIH/TMH*) on -------(*date when participant was recruited*). We would also like to ask you about your overall experience when you were treated in the emergency department at the time of your recruitment into this study.

We will audio recorded this interview and have what you said transcribed just as you said it but without any information that could identify you or others to protect your confidentiality. This audio recording will be securely stored for data analysis purposes only and destroyed as soon as is possible after the analysis is complete. At any time, you can refuse to answer any question or chose to end the interview. Do you agree to be interviewed by me today?"

Completing this interview will probably take 45 minutes to an hour of your time. There are a number of questions we would like you to answer y. There are no right or wrong answers, this is about your experiences.

There are no questions that should cause you any discomfort. Your taking part in this research interview is completely voluntary. You are free to choose not to complete or take part in this interview.

Your completion of this interview may not benefit you personally. We are hoping these completed interviews will provide information to help us to understand how the Navigator study could be improved to help others

The interviews from this study will be kept confidential. None of the information you provide will have your name or any number on it that will identify your personally.

If you have any questions about this interview or the research study itself, please feel free to ask the research assistant providing you with this information. Or you can call us at 444-4444.

If you have any questions about your rights as a research subject please feel free to call our Research Protections Office Director, Janice Muratori, at 444-6246.

After the participant has agreed to continue with the interview the researcher will implement the semi-structured interview guide (see Appendices 2 and 3). When the interview is completed the participant will then be compensated and thanked for their involvement; study staff will upload the audio file for transcription, and complete the staff field notes and debriefing questions of the post-interview debriefing form (see Appendix 4) as to capture their perceptions of the overall interview.

Appendix 2: **Interview Guide for Navigator Study: Patient Assigned to Social Work or Peer Navigator Intervention**

[TURN ON TAPE RECORDER; READ]:
PREAMBLE/CONSENT [READ ALOUD, CONSENT MUST BE DOCUMENTED BY RESEARCH ASSISTANT]: Hi, my name is **[interviewer name]** and I/m a member of

the Navigator research team that will be conducting this interview with you today. We want to ask you about your experiences of taking part in the Navigator study when you were treated in the emergency department at (RIH/TMH) on -------(date when participant was recruited). We would also like to ask you about your overall experience when you were treated in the emergency department at the time of your recruitment into this study.

We will audio recorded this interview and have what you said transcribed just as you said it but without any information that could identify you or others to protect your confidentiality. This audio recording will be securely stored for data analysis purposes only and destroyed as soon as is possible after the analysis is complete. At any time, you can refuse to answer any question or chose to end the interview. Do you agree to be interviewed by me today?"

Completing this interview will probably take 45 minutes to an hour of your time. There are a number of questions we would like you to answer verbally. There are no right or wrong answers, this is about your experiences.

There are no questions that should cause you any discomfort. Your taking part in this research interview is completely voluntary. You are free to choose not to complete or take part in this interview.

Your completion of this interview may not benefit you personally. We are hoping these completed interviews will provide information to help us to understand how the Navigator study could be improved to help others

The interviews from this study will be kept confidential. None of the information you provide will have your name or any number on it that will identify your personally.

If you have any questions about this interview or the research study itself, please feel free to ask the research assistant providing you with this information. Or you can call us at 444-4444.

If you have any questions about your rights as a research subject please feel free to call our Research Protections Office Director, Janice Muratori, at 444-6246.

No one outside of this study will have access to these recordings and they will be destroyed after our final report is written. **Just to confirm, is it OK that we record this? Do you consent to participate and agree to proceed? Yes No (document)**

Do you have any questions before we begin?

OK, this is [**interviewer name**] conducting an interview with **[name/ID]** on **[date]** at about **[time].**

SECTION 1: INTRODUCTION

1. **INTRO:** To get started, can you tell me why you wanted to participate in this interview today?

2. **Terms intro:** Those are all good reasons. Now, just so we are on the same page for our discussion today, when I use the terms OPIOID I'll be talking about drugs/ prescription medications like Percocet, oxycodone, heroin, or fentanyl, that can cause an overdose or that people can become dependent on. And when I say NALOXONE or its other name, NARCAN, I'll be talking about the medication that can reverse opioid overdose.
3. **Research assistant:** When you were in the emergency department at (RIH/TMH) **on** (date) you were recruited into the Navigator study. The person who asked you to be part of the study is the research assistant and I'll be mentioning that person as part of this conversation.
4. **Treatment arm:** After you answered questions you were randomized to either see a social worker or a peer navigator, you might call this person the Anchor recovery coach or counselor. Who did you see? (confirm randomization). I'm going to be referring to the social worker or the Anchor person/peer navigator as part of our conversation.

Any questions so far?

SECTION 2: Navigator study participation experience

Please go through what you did with the research assistant in taking part in the Navigator study when you were in the ED, before you talked to the social worker/peer navigator.

Probes: What were some things that you were being asked to do that weren't clear to you?

What are your thoughts about the survey questions you answered? What do you think the survey questions were getting at?

Why do you think you're being asked to keep in contact with the study and answer more survey questions?

What do you get out of being part of this study?

After hearing about the study, did you feel pressured to participate?

Probes (General) : *Tell me more about that.*

How did you feel about that?

What do you mean when you say [xxx]?

SECTION 3a: Treatment arm experience- social work

After you were randomized you were told that a social worker would be talking to you about your opioid use. What was that like to talk to the social worker about your opioid use?

You only talked to the social worker that night, what were your thoughts about talking to the social worker again or anyone else afterwards about using opioids?

What, if anything, did you take away from the talk you had with the social worker?

Probes: What were you thinking and feeling when you were told that you would be speaking to a social worker about your opioid use?
Have you ever talked to a social worker or anyone else about your opioid use or about anything else?
What was different about talking to that social worker that to anyone else you've talked to about your opioid use?
What do you think you got out of that talk compared to the social worker??
When you think back, what do you wish you had to talked to the social worker about but held back from?
Tell me about anything that changed for you about your opioid use after you talked to the social worker?
Describe anything that was being suggested to you about changing your opioid use you were uncomfortable with?
What's your thoughts on patients being offered counseling in the ED about their opioid use?
Are you familiar with Peer Navigators? If so, what have you heard? If you had the choice, would you have preferred to speak with a Peer Navigator?

Probes (General) : *Tell me more about that.*
How did you feel about that?
What do you mean when you say [xxx]?

SECTION 3b: Treatment arm experience- peer navigator

After you were randomized you were told that a peer navigator (*or term participant uses***) would be talking to you about your opioid use. What was that like to talk to the peer navigator (or term that participant uses) about your opioid use?**

What, if anything, did you take away from the talk you had with the peer navigator?

Peer navigators usually stay in contact after a person has left the ED, what's been your contact with the peer navigator?

Probes: What were you thinking and feeling when you were told that you would be speaking to a peer navigator about your opioid use?
Have you ever talked to a peer navigator or anyone else about your opioid use or about anything else?
Have you continued to keep contact with the peer navigator?
What was different about talking to that peer navigator that to anyone else you've talked to about your opioid use?

What do you think you got out of that talk compared to talking to the peer navigator?

When you think back, what do you wish you had to talked to the peer navigator about but held back from?

Tell me about anything that changed for you about your opioid use after you talked to the peer navigator?

Describe anything that was being suggested to you about changing your opioid use you were uncomfortable with?

What's your thoughts on patients being offered counseling in the ED about their opioid use?

Ig given the choice, would have preferred to speak to a social worker over the peer navigator?

Probes (General) : *Tell me more about that.*
How did you feel about that?
What do you mean when you say [xxx]?

SECTION 4: Opioid use treatment in the ED and community

During your time in the ED tell me about your experiences with the doctors, nurses or EMTs around your opioid use.

Did you discusstreatment or help you could get in the ED or in the community for your opioid use?

Have you ever been in treatment for your opioid use? What kind? How do you view your treatment experience(s)?

Probes: Describe the discussion you had with (*probe who this was*).
How did you feel about that discussion?
Describe anything that was being suggested to you about changing your opioid use you were uncomfortable with?
What treatment options were offered, in the ED (MAT?), or elsewhere?

Have you ever been on medication for addiction treatment (methadone, buprenorphine (Suboxone), naltrexone?)

Probes (General) : *Tell me more about that.*
How did you feel about that?
What do you mean when you say [xxx]?

SECTION 5: Naloxone in the ED

I talked about naloxone when we started our conversation.
When you were in the ED, were you offered naloxone to take home with you ?
What were your thoughts about being offered naloxone?

Probes: Before the ED visit had you gotten naloxone before?
(If naloxone offered and given). Tell me why you decided to take naloxone home with you?
(If naloxone not offered). What are your thoughts about not being offered naloxone to take home?
(If naloxone offered and refused). Tell me about your decision not to take naloxone home with you.
Did you have a family member or friend with you?
Did you believe that there would be a cost associated with accepting the naloxone?

Do you carry naloxone?

Probes (General) : *Tell me more about that.*
How did you feel about that?
What do you mean when you say [xxx]?

SECTION 6: Overall ED and treatment experience

Apart from the Navigator study how would you describe your overall ED experience ?
How did that experience influence whether you accepted services in the ED?How did that experience influence your decision to take part in the Navigator study?

Probes:
Where were you seen in the emergency department?
What would have helped you when you were in the emergency department?
Were you worried about anything?
What do you think would benefit people in the emergency department who have had an overdose? What do you think will help people?
What do you think should be priorities when people are treated after an opioid overdose?
Describe anything that you were emotionally uncomfortable with?

Probes (General) : *Tell me more about that.*
How did you feel about that?
What do you mean when you say [xxx]?

SECTION 7: Stigma

Many individuals feel stigmatized by others when they find out they use drugs.
Tell me about your experiences around stigma when people know you use drugs.
What do people do or say that makes you feel stigmatized?
Did you experience any stigma while you were in the emergency department?
Probes:
How do you think your experience of stigma in the emergency department impacted the medical care you received?

How did your experience of stigma impact services provided to you for your opioid use?
How did this impact whether you wanted to accept services offered?
What do you think could have improved your experience?

During the navigator study what experiences did you have of being stigmatized?
What could the ED or Navigator study do differently to make patients feel less stigmatized?

Probes (General) : *Tell me more about that.*
How did you feel about that?
What do you mean when you say [xxx]?

CONCLUDING QUESTION: Is there anything else related to your feelings about the emergency department services or the study that you would like to talk about today?

Thank you for participating in this interview your answers will help us with our study.

Appendix 3: **Interview Guide for Navigator Study: Patient Assigned to Patient Assigned to Control Group**

[TURN ON TAPE RECORDER; READ]:

PREAMBLE/CONSENT [READ ALOUD, CONSENT MUST BE DOCUMENTED BY RESEARCH ASSISTANT]: Hi, my name is **[interviewer name]** and I/m a member of the Navigator research team that will be conducting this interview with you today. We want to ask you about your experiences of taking part in the Navigator study when you were treated in the

emergency department at (RIH/TMH) on -------(date when participant was recruited). We would also like to ask you about your overall experience when you were treated in the emergency department at the time of your recruitment into this study.

We will audio recorded this interview and have what you said transcribed just as you said it but without any information that could identify you or others to protect your confidentiality. This audio recording will be securely stored for data analysis purposes only and destroyed as soon as is possible after the analysis is complete. At any time, you can refuse to answer any question or chose to end the interview. Do you agree to be interviewed by me today?"

Completing this interview will probably take 45 minutes to an hour of your. There are a number of questions we would like you to answer verbally. There are no right or wrong answers, this is about your experiences.

There are no questions that should cause you any discomfort. Your taking part in this research interview is completely voluntary. You are free to choose not to complete or take part in this interview.

Your completion of this interview may not benefit you personally. We are hoping these completed interviews will provide information to help us to understand how the Navigator study could be improved to help others

The interviews from this study will be kept confidential. None of the information you provide will have your name or any number on it that will identify your personally.

If you have any questions about this interview or the research study itself, please feel free to ask the research assistant providing you with this information. Or you can call us at 444-4444.

If you have any questions about your rights as a research subject please feel free to call our Research Protections Office Director, Janice Muratori, at 444-6246.

No one outside of this study will have access to these recordings and they will be destroyed after our final report is written. **Just to confirm, is it OK that we record this? Do you consent to participate and agree to proceed? Yes No (document)**

Do you have any questions before we begin?

OK, this is [**interviewer name**] conducting an interview with **[name/ID]** on **[date]** at about **[time].**

SECTION 1: INTRODUCTION

5. **INTRO:** To get started, can you tell me why you wanted to participate in this interview today?

6. **Terms intro:** Those are all good reasons. Now, just so we are on the same page for our discussion today, when I use the terms OPIOID I'll be talking about drugs/prescription medications like Percocet, oxycodone, heroin, or fentanyl, that can cause an overdose or

that people can become dependent on. And when I say NALOXONE or its other name, NARCAN, I'll be talking about the medication that can reverse opioid overdose.

7. **Research assistant:** When you were in the emergency department at (RIH/TMH) on (date) you were recruited into the Navigator study. The person who asked you to be part of the study is the research assistant and I'll be mentioning that person as part of this conversation.
8. **Control arm:** After you answered questions, you decided not to see either a social worker or a peer navigator, you might also call this person the Anchor recovery coach or counselor. I just want to confirm that when you took part in this study in the ED you did not talk to either a social worker or a peer navigator (confirm control arm). I'm going to be referring to the social worker or the Anchor person/peer navigator as part of our conversation.

Any questions so far?

SECTION 2: Navigator study participation experience

Please go through what you did with the research assistant in taking part in the Navigator study when you were in the ED.

Probes: What were some things that you were being asked to do that weren't clear to you?

What are your thoughts about the survey questions you answered? What do you think the survey questions were getting at?
Why do you think you're being asked to keep in contact with the study and answer more survey questions?
What do you get out of being part of this study?
After hearing about the study, did you feel pressured to participate?

Probes (General): *Tell me more about that.*
How did you feel about that?
What do you mean when you say [xxx]?

SECTION 3: Decision not to take up Navigator treatment offer

Can you tell me about your decision not to talk to social worker or a recovery coach in the ED?

Who else might you have talked to about your opioid use during that ED visit?

Probes: How were you feeling when you were seen in the emergency department?
Were you uncomfortable? In pain? In opioid withdrawal?
What were you thinking and feeling when you were told that you could speak to a social worker or peer navigator about your opioid use?
Have you ever talked to a social worker, recovery coach, or anyone else about your opioid use?
Why did you not want to talk to the social worker or the recovery coach?
Did prior experiences from family and/or friends with receiving either of these services influence your decision?

Did you believe that talking to either a social worker or recovery coach would lengthen your hospital stay and possibly lead to a hospital admission?

When you think back now on the decision you made, what do think?

Since that ED visit, have you talked to anyone about your opioid use?

What are your thoughts on patients being offered counseling in the ED about their opioid use?

What do you think ED counseling around opioid use and opioid overdose should be like?

Probes (General): *Tell me more about that.*
How did you feel about that?
What do you mean when you say [xxx]?

SECTION 4: Opioid use treatment in the ED and community

During your time in the ED tell me about your experiences with the doctors, nurses or EMTs around your opioid use.

Did you discuss treatment or help you could get in the ED or in the community for your opioid use?

Have you ever been in treatment for your opioid use? What kind? How do you view your treatment experience(s)?

Probes: Describe the discussion you had with (*probe who this was*).
How did you feel about that discussion?
Describe anything that was being suggested to you about changing your opioid use you were uncomfortable with?
What treatment options were offered, in the ED (MAT?), or elsewhere?
Have you ever been on medication for addiction treatment (methadone, buprenorphine (Suboxone), naltrexone?)

Probes (General): *Tell me more about that.*
How did you feel about that?
What do you mean when you say [xxx]?

SECTION 5: Naloxone in the ED

I talked about naloxone when we started our conversation.

When you were in the ED, were you offered naloxone to take home with you?

What were your thoughts about being offered naloxone?

Probes: Before the ED visit had you gotten naloxone before?
(If naloxone offered and given). Tell me why you decided to take naloxone home with you?
(If naloxone not offered). What are your thoughts about not being offered naloxone to take home?
(If naloxone offered and refused). Tell me about your decision not to take naloxone home with you.

Did you have a family member or friend with you?
Did you believe that there would be a cost associated with accepting the naloxone?

Do you carry naloxone?

Probes (General): *Tell me more about that.*
How did you feel about that?
What do you mean when you say [xxx]?

SECTION 6: Overall ED and treatment experience

Apart from the Navigator study, how would you describe your overall ED experience?

How did that experience influence whether you accepted services in the ED?

How did that experience influence your decision to take part in the Navigator study?

Probes:
Where were you seen in the emergency department?
What would have helped you when you were in the emergency department?
Were you worried about anything?
What do you think would benefit people in the emergency department who have had an overdose? What do you think will help people?
What do you think should be priorities when people are treated after an opioid overdose?
Describe anything that you were emotionally uncomfortable with?

Probes (General): *Tell me more about that.*
How did you feel about that?
What do you mean when you say [xxx]?

SECTION 7: Stigma

Many individuals feel stigmatized by others when they find out they use drugs. Tell me about your experiences around stigma when people know you use drugs.

What do people do or say that makes you feel stigmatized?

Did you experience any stigma while you were in the emergency department?

Probes: How do you think your experience of stigma in the emergency department impacted the medical care you received?
How did your experience of stigma impact services provided to you for your opioid use?
How did this impact whether you wanted to accept services offered?
What do you think could have improved your experience?
During the Navigator study what experiences did you have of being stigmatized?
What could the ED or Navigator study do differently to make patients feel less stigmatized?

Probes (General): *Tell me more about that.*
How did you feel about that?
What do you mean when you say [xxx]?

CONCLUDING QUESTION: Is there anything else related to your feelings about the emergency department services or the study that you would like to talk about today?

Thank you for participating in this interview your answers will help us with our study and improve emergency department care.

Appendix 4: **Qualitative Interview Debriefing Form**

To be completed by research staff conducting the patient interview.

Interviewer Name:

Date:

Time:

Location:

Participant ID:

General impressions of the overall interview: (Include environmental factors such as noise, distractions; notes on if you covered the full guide, any challenges with the guide or process that can be improved upon, etc.)

Key Take-aways: What was especially interesting about this interview? What surprised you and what did you learn?

Unanswered questions: What did the interview leave you curious to know more about?

Appendix 5: **Qualitative Interview Audio Recordings Protocol**

Uploading Audio:

1) Visiting site of transcription service.

2) Complete the form
    a. Company Name: Rhode Island Hospital
    b. Your email <<lifespan email>>
    c. Project name: <<study name>>

d. Instructions: <<instructions or tips for transcription. **Indicate if the transcriptionist should use generic placeholders.** It is suggested to list commonly referenced phrases, locations, or slang which may be difficult for the transcriptionist to capture. Examples could include: Subes/ Suboxone; boot (i.e. to inject drugs intravenously). You may also list a priority order if uploading multiple files, or indicate that you would like expedited transcription services (for an additional fee)>>

3) Select files using the file uploader on the right hand side of the screen. Drag and drop, or select files and folders. Click the orange "Start Upload" button at the bottom right.

4) There is a progress bar and an ETA to help you gauge the progress of your upload. Wait for confirmation that the files have uploaded. The message will read "# files have successfully finished uploading. The recipient will now be notified of your files arriving." Once this message is received, the upload is complete.

5) Point person forwards the upload notification to the point of contact on the upload form. If you do not receive this confirmation from the point person, please contact transcription service to confirm the package was received.

Note: the file upload is a secured network, but transcripts are returned by regular email. Please indicate in the instructions whether the transcriptionist should us a generic placeholder for names which appear in the audio file.

Receiving Transcriptions:

1) You will receive an email with the transcripts. Save them as password protected documents in the appropriate shared drive.

2) Review the transcripts against the audio file for accuracy. Use track changes.

3) Review every transcript for anonymity (as appropriate for the project). Replace names and specific locations which may be identifying with placeholders like "NAME" or "CITY IN Rhode Island."

4) Format the document appropriately. Confirm that there is continuous line numbers and page numbers in the document. Add the participant number or focus group name to the header. If track changes were used to make edits to transcript content, save a clean copy. Name the files like: name of project transcript_participant ID.

Commonly used words for Dr. Francesca Beaudoin's Studies:

You may use items on this list to help complete the Instructions section of the upload form. Providing the transcriptionist context and jargon or location specific language will help ensure a more accurate transcription.

“Dear transcriptionist, please note that these interview are with people who use drugs, so there may be some jargon related to drug use or local resources. There is a list for your reference below:”

- ED/ER: Emergency Department/Emergency Room
- OD: overdose
- RA: Research Assistant
- Opioid(s): class of medications
- Percocet, Percs: a type of medication
- Oxycodone, Oxys: a type of medication
- Subes/ Suboxone: a type of medication
- Benzo/ Benzodiazapine: a type of medication
- Naloxone: a type of medication
- Narcan: a type of medication
- Peer Navigator/Coach: a type of counselor

REFERENCES CITED

1. Overdose Death Rates. 2017. (Accessed January 7, 2017, at https://www.drugabuse.gov/related-topics/trends-statistics/overdose-death-rates.)
2. Rudd RA, Aleshire N, Zibbell JE, Gladden RM. Increases in Drug and Opioid Overdose Deaths--United States, 2000-2014. MMWR Morbidity and mortality weekly report 2016;64:1378-82.

3. SAMHSA. Drug Abuse Warning Network, 2011: National Estimates of Drug-Related Emergency Department Visits. Rockville, MD: Substance Abuse and Mental Health Services Administration; 2013.
4. Stoové M, Dietze P, Jolley D. Overdose deaths following previous nonfatal heroin overdose: record linkage of ambulance attendance and death registry data. Drug Alcohol Rev 2009;28:347-52.
5. Pavarin RM, Berardi D, Gambini D. Emergency department presentation and mortality rate due to overdose: A retrospective cohort study on nonfatal overdoses. Subst Abus 2016;37:558-63.
6. Gjersing L, Jonassen K, Skurtveit S, Bramness J, Clausen T. Emergency service use is common in the year before death among drug users who die from an overdose. J Subst Use 2017;22:331-6.
7. Wagner KD, Liu L, Davidson PJ, Cuevas-Mota J, Armenta RF, Garfein RS. Association between non-fatal opioid overdose and encounters with healthcare and criminal justice systems: Identifying opportunities for intervention. Drug and alcohol dependence 2015;153:215-20.
8. Samuels E. Emergency department naloxone distribution: a Rhode Island department of health, recovery community, and emergency department partnership to reduce opioid overdose deaths. Rhode Island medical journal 2014;97:38-9.
9. Galanter M, Dermatis H, Egelko S, De Leon G. Homelessness and mental illness in a professional- and peer-led cocaine treatment clinic. Psychiatr Serv 1998;49:533-5.
10. Huselid RF, Self EA, Gutierres SE. Predictors of successful completion of a halfway-house program for chemically-dependent women. The American journal of drug and alcohol abuse 1991;17:89-101.
11. Tracy K, Burton M, Miescher A, et al. Mentorship for Alcohol Problems (MAP): a peer to peer modular intervention for outpatients. Alcohol and alcoholism 2012;47:42-7.
12. Jason LA, Olson BD, Ferrari JR, Lo Sasso AT. Communal housing settings enhance substance abuse recovery. American journal of public health 2006;96:1727-9.
13. Tracy K, Wallace SP. Benefits of peer support groups in the treatment of addiction. Substance abuse and rehabilitation 2016;7:143-54.
14. Manning V, Best D, Faulkner N, et al. Does active referral by a doctor or 12-Step peer improve 12-Step meeting attendance? Results from a pilot randomised control trial. Drug and alcohol dependence 2012;126:131-7.
15. Zemore SE, Kaskutas LA. 12-step involvement and peer helping in day hospital and residential programs. Substance use & misuse 2008;43:1882-903.
16. Rhode Island Overdose Prevention and Intervention Task Force Action Plan. 2017.
17. Recovery coaches at ERs try to help opioid addicts avoid another overdose. 2017. at https://www.washingtonpost.com/national/health-science/recovery-coaches-at-ers-try-to-help-opioid-addicts-avoid-another-overdose/2017/07/21/6568d1d0-602d-11e7-84a1-a26b75ad39fe_story.html?utm_term=.ac809ea58f33.)
18. Strategies to Combat the Opioid Epidemic. 2017. at https://www.povertyactionlab.org/sites/default/files/documents/Final_Opioid%20Brief_1.5.17.pdf.)
19. Rudd RA, Seth P, David F, Scholl L. Increases in Drug and Opioid-Involved Overdose Deaths - United States, 2010-2015. MMWR Morbidity and mortality weekly report 2016;65:1445-52.
20. IMS Institute for Healthcare Informatics. Medicines Use and Spending in the U.S. ‚Äì A Review of 2015 and Outlook to 2020. April 2016. (Accessed 12/6/2016, at http://www.imshealth.com/en/thought-leadership/quintilesims-institute/reports/medicines-use-and-spending-in-the-us-a-review-of-2015-and-outlook-to-2020.)

21. Kertesz SG. Turning the Tide or Riptide? the Changing Opioid Epidemic. Substance abuse : official publication of the Association for Medical Education and Research in Substance Abuse 2016:0.
22. Hirsch A, Proescholdbell SK, Bronson W, Dasgupta N. Prescription histories and dose strengths associated with overdose deaths. Pain medicine (Malden, Mass) 2014;15:1187-95.
23. Wu LT, Zhu H, Swartz MS. Treatment utilization among persons with opioid use disorder in the United States. Drug and alcohol dependence 2016;169:117-27.
24. Dart RC, Surratt HL, Cicero TJ, et al. Trends in opioid analgesic abuse and mortality in the United States. The New England journal of medicine 2015;372:241-8.
25. Vaughn MG, Salas-Wright CP, Oh S. Trends in heroin access among adolescents in the United States, 2002-2014. Prev Med 2017;99:67-8.
26. Meiman J, Tomasallo C, Paulozzi L. Trends and characteristics of heroin overdoses in Wisconsin, 2003-2012. Drug Alcohol Depend 2015;152:177-84.
27. Jones CM, Logan J, Gladden RM, Bohm MK. Vital Signs: Demographic and Substance Use Trends Among Heroin Users - United States, 2002-2013. MMWR Morb Mortal Wkly Rep 2015;64:719-25.
28. Lipari RN, Hughes A. Trends in Heroin Use in the United States: 2002 to 2013. The CBHSQ Report. Rockville (MD)2013.
29. Barrio G, Montanari L, Bravo MJ, et al. Trends of heroin use and heroin injection epidemics in Europe: findings from the EMCDDA treatment demand indicator (TDI). J Subst Abuse Treat 2013;45:19-30.
30. Victor GA, Walker R, Cole J, Logan TK. Opioid analgesics and heroin: Examining drug misuse trends among a sample of drug treatment clients in Kentucky. Int J Drug Policy 2017;46:1-6.
31. Swanson DM, Hair LS, Strauch Rivers SR, et al. Fatalities Involving Carfentanil and Furanyl Fentanyl: Two Case Reports. J Anal Toxicol 2017:1-5.
32. Dwyer JB, Janssen J, Luckasevic TM, Williams KE. Report of Increasing Overdose Deaths that include Acetyl Fentanyl in Multiple Counties of the Southwestern Region of the Commonwealth of Pennsylvania in 2015-2016. J Forensic Sci 2017.
33. Marshall BDL, Krieger MS, Yedinak JL, et al. Epidemiology of fentanyl-involved drug overdose deaths: A geospatial retrospective study in Rhode Island, USA. Int J Drug Policy 2017.
34. Carroll JJ, Marshall BDL, Rich JD, Green TC. Exposure to fentanyl-contaminated heroin and overdose risk among illicit opioid users in Rhode Island: A mixed methods study. Int J Drug Policy 2017.
35. Somerville NJ, O'Donnell J, Gladden RM, et al. Characteristics of Fentanyl Overdose - Massachusetts, 2014-2016. MMWR Morbidity and mortality weekly report 2017;66:382-6.
36. Mercado MC, Sumner SA, Spelke MB, Bohm MK, Sugerman DE, Stanley C. Increase in Drug Overdose Deaths Involving Fentanyl-Rhode Island, January 2012-March 2014. Pain medicine (Malden, Mass) 2017.
37. Bode AD, Singh M, Andrews J, Kapur GB, Baez AA. Fentanyl laced heroin and its contribution to a spike in heroin overdose in Miami-Dade County. The American journal of emergency medicine 2017.
38. Opioid-Related Inpatient Stays and Emergency Department Visits by State, 2009–2014. HCUP Statistical Brief #219. Agency for Healthcare Research and Quality, 2016. (Accessed Dec 18, 2016,
39. Administration SAaMHS. Drug Abuse Warning Network, 2011: National Estimates of Drug-Related Emergency Department Visits. Rockville, MD: Substance Abuse and Mental Health Services Administration; 2013.

40. Cunningham RM, Bernstein SL, Walton M, et al. Alcohol, tobacco, and other drugs: future directions for screening and intervention in the emergency department. Academic emergency medicine : official journal of the Society for Academic Emergency Medicine 2009;16:1078-88.
41. Choo EK, Ranney ML, Aggarwal N, Boudreaux ED. A systematic review of emergency department technology-based behavioral health interventions. Academic emergency medicine : official journal of the Society for Academic Emergency Medicine 2012;19:318-28.
42. Cunningham R, Mello MJ. Clearing the smoke: behavioral interventions in the emergency department. Academic emergency medicine : official journal of the Society for Academic Emergency Medicine 2011;18:635-6.
43. Cherpitel CJ, Korcha RA, Moskalewicz J, Swiatkiewicz G, Ye Y, Bond J. Screening, brief intervention, and referral to treatment (SBIRT): 12-month outcomes of a randomized controlled clinical trial in a Polish emergency department. Alcoholism, clinical and experimental research 2010;34:1922-8.
44. Bernstein E, Bernstein J, Feldman J, et al. An evidence based alcohol screening, brief intervention and referral to treatment (SBIRT) curriculum for emergency department (ED) providers improves skills and utilization. Subst Abus 2007;28:79-92.
45. Affronti M, Affronti A, Pagano S, et al. The health of irregular and illegal immigrants: analysis of day-hospital admissions in a department of migration medicine. Intern 2013;8:561-6.
46. Repper J, Carter T. A review of the literature on peer support in mental health services. J Ment Health 2011;20:392-411.
47. Boisvert R, Martin L, Grosek M, Clarie A. Effectiveness of a peer -support community in addiction recovery: participation as intervention. Occup Ther Int 2008;15:205-20.
48. Deering K, Kerr T, Tyndall M, et al. A peer-led mobile outreach program and increased utilization of detoxification and residential drug treatment among female sex workers who use drugs in a Canadian setting. Drug Alcohol Depend 2011;113:46-54.
49. Treloar C, Rance J, Bath N, et al. Evaluation of two community-controlled peer support services for assessment and treatment of hepatitis C virus infection in opioid substitution treatment clinics: The ETHOS study, Australia. Int J Drug Policy 2015;26:992-8.
50. Bassuk EL, Hanson J, Greene RN, Richard M, Laudet A. Peer-Delivered Recovery Support Services for Addictions in the United States: A Systematic Review. J Subst Abuse Treat 2016;63:1-9.
51. James T, Bibi S, Langlois B, Dugan E, Mitchell P. Boston Violence Intervention Advocacy Program: A Qualitative Study of Client Experiences and Perceived Effect. Acad Emerg Med 2014;21:742-51.
52. Gonzalez SA, Fierer DS, Talal AH. Medical and Behavioral Approaches to Engage People Who Inject Drugs Into Care for Hepatitis C Virus Infection. Addict Disord Their Treat 2017;16:S1-S23.
53. Could peer-recovery coaches help fight drug addiction epidemic? at http://www.cnn.com/2016/10/24/health/peer-recovery-coaches-drug-addition-epidemic/index.html.)
54. Samuels EA. Emergency department naloxone distribution: A Rhode Island Department of Health, recovery community, and emergency department partnership to reduce opioid overdose deaths. RI Med J 2014;91:38-9.
55. Vestal C. Recovery coaches at ERs try to help opioid addicts avoid another overdose. The Washington Post 2017 July 22.
56. Rhode Island's Strategic Plan on Addiction and Overdose: Four Strategies to Alter the Course of an Epidemic. Rhode Island Governor's Overdose Prevention and Intervention Task Force, 2015. (Accessed Oct 12, 2016, at

http://www.health.ri.gov/news/temp/RhodeIslandsStrategicPlanOnAddictionAndOverdose.pdf.)
57. Jiang Y, McDonald JV, Koziol J, McCormick M, Viner-Brown S, Alexander-Scott N. Can Emergency Department, Hospital Discharge, and Death Data Be Used to Monitor Burden of Drug Overdose in Rhode Island? J Public Health Manag Pract 2017;23:499-506.
58. Darke S, Mills K, Ross J, Teesson M. Rates and correlates of mortality amongst heroin users: findings from the Australian Treatment Outcome Study (ATOS), 2001-2009. Drug Alcohol Depend 2011;115:190-5.
59. Prochaska J, Redding C, Evers K. The Transtheoretical Model and Stages of Change. In: K. Glanz BKRFML, ed. Health Behavior and Health Education: Theory, Research, and Practice. 3rd ed. San Francisco, CA2002.
60. Miller WR, & Rollnick, S. , 3rd ed. Guilford Press, 2012. Motivational Interviewing. 3rd ed. New York, NY: Guilford Publications; 2012.
61. Smedslund G, Berg RC, Hammerstrom KT, et al. Motivational interviewing for substance abuse. Cochrane Database Syst Rev 2011:CD008063.
62. Dunn C, Deroo L, Rivara FP. The use of brief interventions adapted from motivational interviewing across behavioral domains: a systematic review. Addiction 2001;96:1725-42.
63. Prochaska J, DiClemente C, Norcross J. In search of how people change: Applications to the addictive behaviors. American Psychologist 1992;47:1102-14.
64. Ball SA, Legow NE. Attachment theory as a working model for the therapist transitioning from early to later recovery substance abuse treatment. Am J Drug Alcohol Abuse 1996;22:533-47.
65. Germain CB, Gitterman A. The life model of social work practice: Advances in theory & practice: Columbia University Press; 1996.
66. Gorey KM, Thyer BA, Pawluck DE. Differential effectiveness of prevalent social work practice models: A meta-analysis. Social Work 1998;43:269-78.
67. Videka-Sherman L. Metaanalysis of research on social work practice in mental health. Social Work 1988;33:325-38.
68. Payne M. Modern social work theory: Oxford University Press; 2015.
69. Reid WJ. Research on task-centered practice. Social Work Research 1997;21:132-7.
70. Hofmann SG, Asnaani A, Vonk IJ, Sawyer AT, Fang A. The Efficacy of Cognitive Behavioral Therapy: A Review of Meta-analyses. Cognit Ther Res 2012;36:427-40.
71. Moore M, Cristofalo M, Dotolo D, et al. When high pressure, system constraints, and a social justice mission collide: A socio-structural analysis of emergency department social work services. Soc Sci Med 2017;178:104-14.
72. Marshall BDL, Yedinak JL, Goyer J, Green TC, Koziol JA, Alexander-Scott N. Development of a Statewide, Publicly Accessible Drug Overdose Surveillance and Information System. American journal of public health 2017;107:1760-3.
73. Prescription Drug Overdose Data & Statistics: Guide to ICD-9-CM and ICD-10 Codes Related to Poisoning and Pain. Centers for Disease Control and Prevention, 2013. (Accessed Jul 27, 2017, at https://www.cdc.gov/drugoverdose/pdf/pdo_guide_to_icd-9-cm_and_icd-10_codes-a.pdf.)
74. McCormick M, Koziol J, Sanchez K. Development and Use of a New Opioid Overdose Surveillance System, 2016. R I Med J (2013) 2017;100:37-9.
75. van Buuren S. Multiple imputation of discrete and continuous data by fully conditional specification. Stat Methods Med Res 2007;16:219-42.
76. White IR, Royston P, Wood AM. Multiple imputation using chained equations: Issues and guidance for practice. Stat Med 2011;30:377-99.

77. Harel O. Inferences on missing information under multiple imputation and two-stage multiple imputation. Statistical Methodology 2007;4:75-89.
78. Lin DY. Proportional means regression for censored medical costs. Biometrics 2000;56:775-8.
79. Lin DY, Wei LJ, Yang I, Ying Z. Semiparametric regression for the mean and rate functions of recurrent events. J Roy Stat Soc B 2000;62:711-30.
80. Merchant RC, Baird JR, Liu T. Short-term Efficacy of a Brief Intervention to Reduce Drug Misuse and Increase Drug Treatment Utilization Among Adult Emergency Department Patients. Academic emergency medicine : official journal of the Society for Academic Emergency Medicine 2015;22:1172-80.
81. Banta-Green CJ, Coffin PO, Merrill JO, et al. Impacts of an opioid overdose prevention intervention delivered subsequent to acute care. Injury prevention : journal of the International Society for Child and Adolescent Injury Prevention 2018.
82. Jiang Y, McDonald JV, Wilson ME, et al. Rhode Island Unintentional Drug Overdose Death Trends and Ranking - Office of the State Medical Examiners Database. Rhode Island medical journal 2018;101:33-6.

Analysis Plan for the Navigator Trial
08/07/2020

I. Study Design

The purpose of this study is to determine the effectiveness of peer navigation versus a standard behavioral intervention delivered in the emergency department (ED) to overdose patients and those at risk of recurrent opioid overdose. A total of 650 ED patients will be recruited from two emergency departments in a single health care system in Providence, Rhode Island into a two-arm randomized trial with 18 months of follow-up post-randomization. Eligible participants will be randomly assigned (1:1) in the ED to receive a behavioral intervention from a certified peer recovery support specialist or a licensed clinical social worker (LCSW). Effectiveness will be measured objectively through linkage to administrative statewide databases, with two primary endpoints: (1) engagement in formal addiction treatment (e.g., inpatient services, outpatient services, medication-assisted treatment [MAT]) from a licensed substance abuse treatment provider within 30 days following the index ED visit; and (2), recurrent ED visit for an opioid overdose within 18-months following the index ED visit. Exploratory outcomes of interest are: overdose fatality, repeat ED visits related to opioids, and successful completion of an addiction treatment program and/or long-term retention in MAT.

II. Research Questions

This study addresses the following research questions:

1. Does peer navigation result in greater early treatment engagement and reduction in recurrent opioid overdose compared to a standard intervention delivered by a clinical social worker among persons presenting to the ED for on opioid overdose or are being treated for a visit related to illicit opioid use?

2. If peer navigation is found to be more effective than standard of care, is there heterogeneity of treatment effect related to key patient characteristics (e.g., sex, race, type of opioid used, and history of comorbid chronic pain, depression or posttraumatic stress disorder)?

III. Study Sample

A total of 650 patients treated for an opioid overdose or at risk for an opioid overdose (defined below) will be recruited (n=325 per arm) from two emergency departments in a single health care system in Providence, Rhode Island and followed prospectively using administrative datasets. Our primary outcomes will be: (1) engagement in treatment within 30-days after the ED visit, and (2) recurrent ED visit for opioid overdose over the 18-month follow-up period.

We will recruit adult ED patients who are: (1) being treated for an opioid overdose, or (2) have had an opioid overdose in the past 12 months (identified by self-report during screening or in review of the EMR); or (3) are presenting with a visit related to illicit injection opioid use (e.g., cutaneous injection-related infection, opioid withdrawal, endocarditis). We are specifically

targeting patients with a current or recent opioid overdose and those who inject opioids illicitly, as they are at highest risk for opioid overdose and death.

All participants will be recruited from two EDs—Level 1 and Level 2 trauma centers—located in the state's capital of Providence. Together, these two EDs receive over 175,000 adult visits each year. Between 2017 and 2018, the two EDs reported a total of 1,446 visits for suspected opioid overdoses, representing 45% of all ED visits for suspected opioid overdoses reported to the Rhode Island Department of Health (n = 3,239).

A consecutive sample of ED patients will be assessed for eligibility by one of eleven full-time research assistants employed in the two EDs who can recruit participants 24 hours per day, seven days a week. Potential participants will be identified by the research assistants by screening electronic medical records (EMR) or by referrals from treating providers in the ED. Patients who meet the initial eligibility screen will undergo a further in-person assessment by a study RA.

Participants will be eligible if they are: (1) English-speaking, (2) 18 years of age or older, and (3) are being treated for an opioid overdose *or* identified as having had an opioid overdose in the past 12 months *or* are being treated for a visit related to illicit opioid use (e.g., abscess, opioid withdrawal), (4) and are able to provide informed consent. Participants are ineligible if they are critically ill or injured, are previously enrolled in the trial, in police custody or incarcerated, pregnant, or live outside Rhode Island (patients must live in state to link to administrative databases). Patients who are critically ill will be eligible once cleared by their physician.

After screening, if the patient is eligible and willing to participate, then full written informed consent will be obtained. After obtaining consent, participants will be randomly assigned 1:1 within each study site to receive either a behavioral intervention delivered by a certified peer recovery support specialist or by an LCSW. Allocations will be randomly assigned using the REDCap randomization feature. The randomization schedule will be maintained by a data manager not involved with participant recruitment or the final study analyses. We anticipate the intervention will begin within 30 minutes of randomization.

Participants and providers cannot be blinded to their intervention assignment, however investigators and analysts performing the study analyses will be blinded to arm allocation.

Throughout the study, participants will remain assigned to the treatment arm to which they were assigned for the purposes of intention-to-treat (ITT) analyses. Following randomization, we will take additional steps to minimize crossover in order to maximize internal validity, and also mimic what would happen if emergency departments only had clinical social workers or only had peer navigators, respectively. Practically speaking, this means that if a participant who is randomized to receive an intervention has a repeat ED visit in which a social worker or a peer navigator would be called, they will be assigned to receive the same intervention on the repeat visit. This is ethically sound as it is consistent with current standard of care. This is feasible for several reasons. First, our electronic medical record (EMR) system allows us to create "flags" for participants enrolled in research studies. This would generate an on-screen notification to the treating providers and also alert the study team via a text notification. Second, our research

assistants are available 24 hours a day, seven days a week to actively assess for repeat visits among study participants during the course of regular patient screening, and will intervene if necessary to ensure a participant receives the same intervention as that which they were assigned at randomization, Third, our hospital system cares for almost 75% of the overdoses in the state, meaning that these notifications will have a broad catchment area. Moreover, a review of data from the state and hospital systems shows that patients tend to return to the same ED when they have a repeat visit. Finally, since the primary outcome of repeat ED visit for overdose requires an ED visit, the outcome will occur before a subsequent intervention (and potential cross-contamination) in most cases. Currently it is only standard of care for patients to receive a behavioral intervention after an overdose, but not necessarily other opioid-related visits. We will measure any protocol deviations through our robust data sources and hospital-based tracking systems (see below).

IV. Data Sources

This RCT will have two primary endpoints: (1) engagement in a formal addiction treatment program from a licensed substance abuse treatment provider within 30 days following the index ED visit; and (2), recurrent ED visit for an opioid overdose within 18 months from the index ED visit. We will obtain objective assessments of these outcomes through the use of statewide administrative database as outlined below:

30-day treatment engagement (primary endpoint 1): The first primary outcome, engagement in addiction treatment, will be defined as the proportion who are admitted to a formal addiction treatment program within thirty days following the initial ED visit. This outcome was chosen because a key short-term goal of the ED behavioral intervention is to promote early treatment engagement. This outcome will be assessed using Rhode Island Department of Behavioral Health, Developmental Disabilities, and Hospitals (BHDDH) and Prescription Drug Monitoring Program (PDMP) records. The BHDDH database contains information on all admissions to publicly funded substance abuse treatment programs in the state. We will define treatment engagement as admission to any of the program types licensed by BHDDH, including inpatient detoxification, day treatment programs, residential treatment, intensive outpatient services, and opioid treatment programs (i.e., methadone). Second, we will query the participant's RI PDMP records in order to identify enrollment in office-based buprenorphine therapy. The RI PDMP manages a database that contains information on all prescriptions for schedule II-IV substances filled in the state. The database is updated daily; all pharmacies are required to report prescriptions within 48-hours of the fill date. The database includes information on the patient (e.g., name, sex, birth date, address including zip code), the prescription filled (e.g., quantity, days supply, national drug code number), and prescriber/pharmacy data. Pharmacies are required by law to report prescriptions to the PDMP regardless of payment type. All records will be linked deterministically to participant data using identifiable information (e.g., name, social security number) within the Stronghold computing environment, a HIPAA-compliant server maintained by Dr. Marshall's team at Brown University. Our research team has experience extracting and analyzing PMDP and BHDDH data for statewide surveillance purposes.

Recurrent ED visits for overdose (primary endpoint 2): The second primary outcome, recurrent ED visit for overdose, will be defined as the proportion of participants who are treated in any Rhode Island ED for an opioid overdose at any time during the 18-month follow-up period following the initial ED visit. Recurrent ED visits for opioid overdose were chosen as the second primary outcome as a long-term goal of the ED behavioral interventions is to reduce fatal and non-fatal overdose. Two data sources will be used to assess this outcome. First, we will access the electronic medical records (EMRs) of the 12 EDs in Rhode Island through the Rhode Island Quality Institute Statewide Health Information Exchange. This data source will be made accessible through Brown's Advance- CTR Unified Research Data Sharing Access (URSA) infrastructure. This unified data system provides access to EMR data from all major health systems in Rhode Island. Thus, we will capture repeat visits for an opioid overdose that occur in all 12 EDs in Rhode Island. We will define an ED visit for an opioid overdose based on CDC guidelines for all opioid poisonings (which includes illicit opioids) and utilizes International Classification of Disease (ICD) coding. Second, we will query the RI Department of Health (RIDOH) Opioid 48-Hour Overdose Surveillance System. The RIDOH mandates all suspected opioid overdose cases presenting to an RI hospital be reported to the department within 48 hours. This data source will capture recurrent overdoses not identified by ICD codes in the unified EMR data system, and also contains additional fields of interest (e.g., pre-existing risk factors for overdose).

Finally, we will determine mortality outcomes by requesting data from the National Death Index (NDI), which is a centralized database of death record information on file in state vital statistics offices. We will use the NDI data in conjunction with the Rhode Island Department of Health medical examiner data to determine whether or not a participant in the study has died during follow-up, and if so, the cause of death (including overdose).

V. Methods

For the two primary outcomes (engagement in formal SUD treatment within 30 days of the initial ED visit and occurrence of a subsequent ED visit for opioid overdose within 18 months of the initial ED visit), we will use separate logistic regression models with indicators for treatment allocation and study site, as well as term representing the interaction of treatment allocation with study site. Second, we will conduct subgroup analyses to understand potential heterogeneity of treatment effects by age and gender.

For all analyses, routine procedures will first be conducted to ensure data accuracy/adequacy. We will use an intention-to-treat (ITT) approach in all analyses to address potential problems inherent in following only intervention completers; a sensitivity analysis ("per protocol") will be conducted among only those that complete the ED intervention. Given use of administrative data sources we anticipate minimal missingness in our final dataset. However, missing outcome and covariate data will be handled using case-wise deletion. Additionally, we will perform a sensitivity analysis to determine the potential impact of missing data on treatment effect. This sensitivity analysis will use multiple imputation performed using chained equations that specify the conditional models for all of the variables with missing values.

Effectiveness, 30-day engagement in treatment (primary endpoint 1): We will compare the effectiveness of the peer navigation versus social work intervention on increasing engagement in formal addiction treatment within 30 days of the initial ED visit. As the primary analysis, we will compare the proportion who are admitted to a licensed addiction treatment program (using chi-square analysis) between the two groups. In the next stage of our primary analysis, logistic regression models will be used to determine the independent effect of the intervention arm on 30-day treatment admission, adjusting for study site and an interaction term between treatment allocation and study site, as described above.

Effectiveness, recurrent ED visit for an opioid overdose (primary endpoint 2): We will compare the effectiveness of the peer navigation versus the social work intervention on preventing subsequent ED visits for opioid overdose. As the primary analysis, we will compare the overall proportion of patients experiencing a subsequent opioid overdose over the 18-month follow-up period between the intervention groups (using chi- square analysis). In the next stage of our primary analysis, logistic regression models will be used to determine the independent effect of the intervention arm on recurrent ED visits for opioid overdoses, adjusting for study site, as described for primary endpoint 1.

In a sensitivity analysis, we will assess imbalance in key prognostic factors for the study outcomes (e.g., lifetime history of overdose, lifetime treatment engagement, age, sex, race, and housing status) between the two study arms. If imbalance is observed, we will include these covariates in the logistic regression models to determine the robustness of the primary analysis results.

In exploratory analyses, we will also examine a number of other outcomes, including: overdose *rates*, overdose death, and successful completion of or retention in addiction treatment. Successful completion of and/or retention in addiction treatment will be defined based on discharge data collected in BHOLD and prescription refill data in the PDMP (e.g., on MAT for ≥6 months). We will examine the time to ED visit for an opioid overdose using a Kaplan-Meier analysis. Patients will be censored at the end of the 18-month follow-up period, considered the last point of contact. We will use Breslow’s method to test if the time to subsequent opioid overdose rates differs between the groups. Next, Cox proportional hazards modeling will be used to estimate hazard ratios (HRs) and corresponding 95% confidence intervals (CIs) for occurrence of repeat overdose between groups. HRs will be adjusted for clinical and demographic characteristics believed to predict the outcome of opioid overdose in order to adjust for possible residual confounding and treatment-factor interactions. Finally, since participants may experience multiple opioid overdoses during follow-up, we will also conduct recurrent-event survival analyses. These models extend the Cox model approach and allow for estimation of hazard ratios pooled across repeated periods at risk. Finally, we will examine if heterogeneity of intervention effect is modified by age, sex, race, pre-existing chronic pain, past treatment history, and reason for presentation to the ED. We will perform stratified subgroup analyses to determine if treatment effects vary between groups of individuals.

Feasibility and Sample Size Calculation. First, we assume that ~2,000 patients will be treated at the two EDs for an opioid overdose over the 24-month recruitment period; this is based on data from the RI Opioid Overdose Surveillance System and is a conservative estimate that does

not include other eligibility criteria (e.g., recent overdose). Next, we assume that 65% (n=1,300) of these patients will be willing to be screened (prior studies of behavioral interventions for drug use at the two EDs have had screening rates > 80%) (Merchant, Baird, & Liu, 2015). Third, we assume that ~50% (n=650) of these patients will be eligible and randomized to an intervention arm (n=325 per arm). Thus, we estimate that 24 months will be required to recruit 650 participants. Given our use of objective outcome data from administrative datasets, we do not expect dropout to significantly impact our statistical power, but our power calculations conservatively reflect a 10% loss to follow-up rate.

For our sample size calculation, we assumed that 7% of participants in the social work arm will enroll in a formal treatment program within 30 days of ED discharge (based on preliminary data from the state and RIH). Given this, we have >80% power to detect a two-fold increase (i.e., >7 percentage point absolute increase) in the rate of 30-day treatment engagement between the two arms (Figure 2A); this increase has been deemed a bench-mark by key state stakeholders. For primary endpoint 2 (recurrent ED visit for an opioid overdose), we assumed that 15% of patients in the social work arm will have a recurrent ED visit for an opioid overdose within 18-months of their first visit. This estimate is based on a chart review of 374 patients after program implementation of the RIH ED behavioral intervention program and in recently published data by Banta-Green et al.(Banta-Green et al., 2018) The latter study from Washington State found around a 20% incidence of overdose within 18-months following of an initial ED visit. Our assumption of a 15% incidence of repeat ED visit for overdose is conservative compared to this finding, particularly in light of the fact the Rhode Island has nearly twice as many overdose deaths per capita than Washington State.(Jiang et al., 2018) We will have >80% power to detect a 50% relative reduction (7.5 percentage point absolute reduction) in the risk of recurrent overdose within 18 months of their ED visit (Figure 2B), this reduction was felt to be clinically relevant and commensurate with statewide goals in reducing overdose via various strategies.

We chose to evaluate the outcome date within the first 18 months after the initial ED visit for two main reasons. First, the risk of recurrent overdose appears to level-off by about 18 months, meaning that most individuals who will experience another overdose will do so within the first 18 months. Second, given the urgent need to have an evidence-based evaluation of peer-led behavioral interventions for OUDs, a shorter length of follow-up would allow us to disseminate our study findings sooner. However, we recognize that there is potential value in having a longer follow-up period both from an impact standpoint and also in terms of statistical power to detect a difference between the treatment groups. Therefore, as a contingency plan, we could extend the length of follow-up by one year (30 months total) based on 18-month outcome analyses. We will make this decision in conjunction with the LJA Foundation.

VI. Correspondence with Ethical Standards for Research

The research protocol has be reviewed and approved by the Lifespan Institutional Review Boards (IRBs) at Rhode Island Hospital (IRB 1 registration: 00000396, IRB 2 registration: 00004624) and The Miriam Hospital (IRB registration 00000482), with approval number 212418.

Banta-Green, C. J., Coffin, P. O., Merrill, J. O., Sears, J. M., Dunn, C., Floyd, A. S., . . . Donovan, D. M. (2018). Impacts of an opioid overdose prevention intervention delivered subsequent to acute care. *Inj Prev*. doi:10.1136/injuryprev-2017-042676

Jiang, Y., McDonald, J. V., Wilson, M. E., Koziol, J., Goldschmidt, A., King, E., . . . Alexander-Scott, N. (2018). Rhode Island Unintentional Drug Overdose Death Trends and Ranking - Office of the State Medical Examiners Database. *R I Med J (2013), 101*(1), 33-36.

Merchant, R. C., Baird, J. R., & Liu, T. (2015). Short-term Efficacy of a Brief Intervention to Reduce Drug Misuse and Increase Drug Treatment Utilization Among Adult Emergency Department Patients. *Acad Emerg Med, 22*(10), 1172-1180. doi:10.1111/acem.12767
