# Supplementary material for: Effect of a Peer-Led Behavioral Intervention for Emergency Department Patients at High Risk of Fatal Opioid Overdose: A Randomized Clinical Trial
Source: JAMA Netw Open. 2022 Aug 9;5(8):e2225582. doi: 10.1001/jamanetworkopen.2022.25582 (PMC9364125; doi:10.1001/jamanetworkopen.2022.25582)
Supplement: Supplement 2. — eTable 1. Enrollment in Substance Use Disorder Treatment Within 30 Days of Enrollment in the Navigator Trial eTable 2. Enrollment in Substance Use Disorder Program Within 30 Days of Enrollment in the Navigator Trial, Stratified by Reason for Emergency Department Visit and Treatment History [file jamanetwopen-e2225582-s002.pdf]

Supplemental Online Content

Beaudoin FL, Jacka BP, Li Y, et al. Effect of a peer-led behavioral intervention for emergency department patients at high risk of fatal opioid overdose: a randomized clinical trial. *JAMA Netw Open*. 2022;5(8):e2225582. doi:10.1001/jamanetworkopen.2022.25582

eTable 1. Enrollment in Substance Use Disorder Program Within 30 Days of Enrollment in the Navigator Trial

eTable 2. Enrollment in Substance Use Disorder Program Within 30 Days of Enrollment in the Navigator Trial, Stratified by Reason for Emergency Department Visit and Treatment History

This supplemental material has been provided by the authors to give readers additional information about their work.

© 2022 Beaudoin FL et al. *JAMA Network Open.*

eTable 1. Enrollment in Substance Use Disorder Program Within 30 Days of Enrollment in the Navigator Trial

Enrollment in	Total [n (%)] (n=648)	Study arm [n (%)]		Model 1[1]		Model 2[2]	
		Social Worker (n = 325)	Peer (n = 323)	Relative risk (IQR)	p-value	Relative risk (IQR)	p-value
No	447 (69)	227 (70)	220 (68)	1.00		1.00	
Yes	201 (31)	98 (30)	103 (32)	1.06 (0.84, 1.33)	0.633	1.05 (0.84, 1.32)	0.673

1 Model 1: unadjusted log-binomial regression; 2 Model 2: Log-binomial regression adjusting for block randomization variables (age </> 50 years; sex at birth; hospital site).

eTable 2. Enrollment in Substance Use Disorder Program Within 30 Days of Enrollment in the Navigator Trial, Stratified by Reason for Emergency Department Visit and Treatment History

	Total [n (%) (n=648)]	Study arm		p-value
		Social Worker [n (%) (n = 325)]	Peer [n (%) (n = 323)]	
**A. Stratified by Reason for the ED Visit**				
Opioid overdose-related visit				
Enrollment in SUD program				
No	212 (72)	106 (74)	106 (71)	0.637
Yes	81 (28)	38 (26)	43 (29)	
Total	293 (100)	144	149	
Non-Opioid overdose-related visit				
Enrollment in SUD program				
No	234 (66)	121 (67)	113 (65)	0.705
Yes	119 (34)	59 (33)	60 (35)	
Total	353 (100)	180	173	
**B. Stratified by Prior Treatment History**				
Prior treatment exposure, past 30 days				
Enrollment in SUD program				
No	80 (45)	42 (48)	38 (43)	0.457
Yes	96 (55)	45 (52)	51 (57)	
Total	176 (100)	87	89	
No prior treatment exposure, past 30 days				
Enrollment in SUD program				
No	367 (78)	185 (78)	182 (78)	0.990
Yes	105 (22)	53 (22)	52 (22)	
Total	472 (100)	238	234	

© 2022 Beaudoin FL et al. *JAMA Network Open.*
