# Supplementary material for: Effect of a Peer-Led Behavioral Intervention for Emergency Department Patients at High Risk of Fatal Opioid Overdose: A Randomized Clinical Trial
Source: JAMA Netw Open. 2022 Aug 9;5(8):e2225582. doi: 10.1001/jamanetworkopen.2022.25582 (PMC9364125; doi:10.1001/jamanetworkopen.2022.25582)
Supplement: Supplement 3. — Data Sharing Statement [file jamanetwopen-e2225582-s003.pdf]

Data Sharing Statement

Beaudoin. Effect of a Peer-Led Behavioral Intervention for Emergency Department Patients at High Risk of Fatal Opioid Overdose. *JAMA Netw Open*. Published August 09, 2022. doi:10.1001/jamanetworkopen.2022.25582

Data
Data available: Yes
Data types: Deidentified participant data
How to access data: Made available by request to the investigative team, please email: francesca_beaudoin@brown.edu
When available: beginning date: 08-01-2023

Supporting Documents
Document types: None

Additional Information
Who can access the data: Researchers whose proposed use of the data has been approved.
Types of analyses: For non-commercial research only.
Mechanisms of data availability: After approval of a proposal and with a signed data use agreement.
